# Skeletal muscle mitochondrial bioenergetics and associations with *myostatin* genotypes in the Thoroughbred horse

**DOI:** 10.1371/journal.pone.0186247

**Published:** 2017-11-30

**Authors:** Mary F. Rooney, Richard K. Porter, Lisa M. Katz, Emmeline W. Hill

**Affiliations:** 1 UCD School of Agriculture and Food Science, University College Dublin, Belfield, Dublin 4, Ireland; 2 School of Biochemistry and Immunology, Trinity Biomedical Sciences Institute (TBSI), Trinity College Dublin, Dublin 2, Ireland; 3 UCD School of Veterinary Medicine, University College Dublin, Belfield, Dublin 4, Ireland; Cinvestav-IPN, MEXICO

## Abstract

Variation in the *myostatin* (*MSTN*) gene has been reported to be associated with race distance, body composition and skeletal muscle fibre composition in the horse. The aim of the present study was to test the hypothesis that *MSTN* variation influences mitochondrial phenotypes in equine skeletal muscle. Mitochondrial abundance and skeletal muscle fibre types were measured in whole muscle biopsies from the *gluteus medius* of n = 82 untrained (21 ± 3 months) Thoroughbred horses. Skeletal muscle fibre type proportions were significantly (p < 0.01) different among the three *MSTN* genotypes and mitochondrial content was significantly (p < 0.01) lower in the combined presence of the C-allele of SNP g.66493737C>T (C) and the SINE insertion 227 bp polymorphism (I). Evaluation of mitochondrial complex activities indicated higher combined mitochondrial complex I+III and II+III activities in the presence of the C-allele / I allele (p ≤ 0.05). The restoration of complex I+III and complex II+III activities following addition of exogenous coenzyme Q_1_ (ubiquinone_1_) (CoQ_1_) *in vitro* in the TT/NN (homozygous T allele/homozygous no insertion) cohort indicated decreased coenzyme Q in these animals. In addition, decreased gene expression in two coenzyme Q (CoQ) biosynthesis pathway genes (*COQ4*, p ≤ 0.05; *ADCK3*, p ≤ 0.01) in the TT/NN horses was observed. This study has identified several mitochondrial phenotypes associated with *MSTN* genotype in untrained Thoroughbred horses and in addition, our findings suggest that nutritional supplementation with CoQ may aid to restore coenzyme Q activity in TT/NN horses.

## Introduction

*Myostatin* knockout mice have a marked increase in muscle mass due to hypertrophy and hyperplasia [1]. Muscle fibre diameter and fibre number are significantly increased in *Mstn*-null mice compared to wildtype littermates. Depending on the method of myostatin inhibition, differing effects are observed; complete knockout induces hypertrophy and hyperplasia whereas partial inhibition results in hypertrophy alone [2]. Myostatin inhibition-induced muscle hypertrophy has been reported to affect slow and fast-twitch muscle fibres equally [3, 4]. However, Girgenrath *et al*. [5] reported a higher proportion of fast-twitch fibres in *soleus* and extensor *digitorum longus* muscles of *Mstn*-null mice than wildtype littermates suggesting that myostatin inhibition may alter skeletal muscle fibre composition.

In addition to increased skeletal muscle mass, *Mstn*-null mice have a lower mitochondrial number and, consequently, an alteration in the activity and enzymatic capacity of the skeletal muscle [6, 7]. Decreased mitochondrial number and/or mitochondrial activity may impact on skeletal muscle function by impairing mitochondrial metabolism and thereby reducing the contractile ability of the muscle. Mitochondria are the major energy transducers in the skeletal muscle and skeletal muscle is the site of most of the body’s oxygen consumption during exercise [8]; therefore, an impairment of mitochondrial function may alter the athletic phenotype.

The Thoroughbred is a horse breed best known for its use in racing. Their origin stems from a narrow founding gene pool, which can be traced back to a small number of Arab, Barb and Turk stallions and native British mares, approximately 300 years ago [9–11]. Intense selection for speed and stamina has led the Thoroughbred to have a very high aerobic capacity relative to skeletal muscle mass [12–15] and as a consequence their agility and speed surpass many other domestic breeds. Although the complete knockout of myostatin in horses has not been investigated, natural genetic variation within the *MSTN* gene has been identified. Variation at SNP g.66493737C>T (hereafter ‘SNP’) in the first intron and a 227 bp SINE insertion polymorphism in the promotor region (Chr18:g.66495326_66495327ins227; hereafter ‘SINE insertion’) has been shown to be associated with optimum race distance in Thoroughbred horses in a number of studies [16–20]. Additionally, the SNP has been observed to have an effect on the body composition of Thoroughbred horses after a period of training, with CC genotype horses having a significantly greater body mass than CT or TT horses [21]. Furthermore, a significant association between the SINE insertion and muscle fibre composition in Thoroughbred and Quarter Horses has been detected [22, 23]. In the Thoroughbred, the presence of the SINE insertion (I allele) was associated with a 6% increase in type IIX fibres and a corresponding 6% decrease in type I fibres, in comparison to no SINE insertion (N allele).

Equine skeletal muscle is a heterogeneous tissue made up of different fibre types denoted type I, type IIA and type IIX [24]. Type I fibres are oxidative, slow-twitch fibres with high mitochondrial and capillary density. In contrast, type IIX fibres are glycolytic, fast-twitch fibres with low mitochondrial and capillary density. Type IIA fibres are fast-twitch, intermediate in their oxidative and glycolytic capacities and have a high mitochondrial density. In that regard, the association with muscle fibre proportion is consistent with the association with best race distance, as the CC genotype horses are best suited to short distance sprint racing requiring short bursts of speed that may be facilitated by fast-twitch muscle fibres and TT genotype horses are best suited to longer distance races, requiring more stamina, and thus a higher oxidative capacity which may be achieved by a higher proportion of type I and type IIA fibres.

The aim of this study was to test the hypothesis that *MSTN* genotype (g.66493737C>T SNP and SINE insertion 227 bp polymorphism) may influence mitochondrial abundance and energetics assessed by the activity of the electron transport chain complexes in Thoroughbred horse skeletal muscle.

## Materials and methods

### Reagents

All reagents and chemicals used were of analytical grade where available and were obtained from Sigma Aldrich Co. LLC., Vale Rd, Arklow, Co. Wicklow, Ireland, unless otherwise stated.

### Animals, ethics and licencing

The study subjects were n = 82 Thoroughbred horses (n = 37 CC/II [15 male, 22 female], n = 34 CT/IN [15 male, 19 female], n = 11 TT/NN [1 male, 10 female]), subsets of which were used for various experimental analysis. Thoroughbred horses (21 ± 3 months) were in the early stages of training (‘untrained’). All horses were considered ‘untrained’ as they had not yet completed any high-intensity exercise (known as a ‘work day’) but had participated in varied amounts of submaximal training, consisting of walking, trotting and cantering (S2 Fig).

Horses were maintained in the same training yard under the supervision and management of a single trainer. All procedures and veterinarians performing the procedures were approved and licenced under the Department of Health/Irish Medicines Board/Health Products Regulatory Authority (HPRA), ethics approval was granted by UCD Animal Research Ethics Committee and owners consent was given.

### Skeletal muscle biopsy sampling

Skeletal muscle biopsies were taken from the middle gluteal muscle of a standing unsedated horse by a qualified and experienced veterinarian as per the techniques described by Ledwith and McGowan [25]. Biopsy samples were stored on dry ice for transport to the laboratory and subsequently stored at -70°C.

### *MSTN* genotyping

#### *MSTN* SNP g.66493737C>T genotyping

Horses were genotyped for the *MSTN* SNP g.66493737C>T by Equinome Ltd. (now part of Plusvital Ireland Ltd.) (http://www.equinome.com/) as per the method described by Hill *et al*. [26]. Briefly, genomic DNA was extracted from fresh whole blood using the Maxwell 16 automated DNA purification system (Promega, WI) and SNP g.66493737C>T genotyping was performed using Taqman chemistry. The Taqman assay mixture contained forward primer 5’-GAC ACA ACA GTT TCA AAA TAT TGT TCT CCT T-3’, reverse primer 5′-CCA GGA CTA TTT GAT AGC AGA GTC A-3’ and two allelic-specific fluorescent dye labeled probes: VIC-AAT GCA CCA AGT AAT TT and 6-FAM-ATG CAC CAA ATA ATT T.

#### *MSTN* SINE insertion 227 bp polymorphism genotyping

Two polymerase chain reaction (PCR)-based assays were used to genotype horses for the *MSTN* SINE insertion 227 bp polymorphism using GoTaq Hot start master mix (Promega) and associated reagents. Primers were used at a final concentration of 0.5 μM. The primer sequences were as follows: Primer set 1; 5’-ATC AGC TCA CCC TTG ACT GTA AC-3’ (forward), 5’-TCA TCT CTC TGG ACA TCG TAC TG-3’ (reverse) [18]; Primer set 2; 5’-ATC AGC TCA CCC TTG ACT GTA AC-3’ (forward), 5’-GTA TTC TTC GTT GTG GGT TCC TC-3’ (reverse). Both assays were run simultaneously using the same PCR conditions, as follows: initial denaturation at 95°C for 5 minutes, followed by 40 cycles of 95°C for 30 seconds, 58°C for 30 seconds and 72°C for 1 minute, followed by a final elongation at 72°C for 5 minutes. The resulting amplification products were electrophoresed on a 2% agarose gel in order to determine the genotype of the animals.

### Measurement of enzyme activities

#### Spectrophotometric enzyme activity assays

All enzyme activity assays were performed at 30°C on a Biochrom Libra S12 spectrophotometer or a UV-2600 UV-Vis spectrophotometer (Shimadzu) and absorbance changes were measured using an attached chart recorder or accompanying UV probe software (Shimadzu), respectively. The activity of each enzyme was measured in at least duplicate on the same homogenate for each sample.

#### Preparation of skeletal muscle homogenates

Skeletal muscle homogenates were prepared from frozen tissue which was previously stored at -70°C. Any fat or connective tissue was removed from the sample and it was weighed using a fine balance (ME104, Mettler Toledo, 0.08 mg repeatability). The tissue was homogenised using an Ultra Turrax T25 (Janke & Kunkel IKA-Labortechnik) in sucrose muscle homogenisation buffer (20 mM tris-HCl, 40 mM KCl, 2 mM EGTA, 250 mM sucrose, pH 7.4). An aliquot of the sample was used to perform protein determination using the Bicinchoninic Acid (BCA) Assay as described by Smith *et al*. [27].

#### Citrate synthase activity assay

Citrate synthase enzyme activity was measured spectrophotometrically by a coloured coupled reaction, by a method adapted from that originally described by Srere [28]. The activity of citrate synthase was determined by monitoring the rate of production of thionitrobenzoic acid (TNB) at a wavelength of 412 nm. Skeletal muscle homogenate (approximately 5 μg) was incubated in a 1 ml cuvette with tris buffer (0.2 M, pH 8.1) and the following reaction components were added; 5,5'-dithiobis-(2-nitrobenzoic acid) (DTNB) (0.1 mM), acetyl coenzyme A (0.3 mM) and Triton X (0.1%). A blank rate was measured for 2 minutes before oxaloacetate (0.5 mM) was added to initiate the reaction and an increase in absorbance was monitored for 3 minutes.

#### NADH-ubiquinone oxidoreductase (Complex I) activity assay

The activity of NADH-ubiquinone oxidoreductase (Complex I) was determined by monitoring the oxidation of NADH at 340 nm, by a method adapted from that described by Ragan *et al*. [29]. Whole muscle homogenate samples were diluted in hypotonic buffer (25 mM potassium phosphate (pH 7.2), 5 mM MgCl_2_) and subjected to three freeze thaw cycles in liquid nitrogen immediately prior to being assayed. Homogenate samples (approximately 20 μg) were incubated in a 1 ml cuvette which contained an assay mixture consisting of potassium phosphate pH 7.5 (50 mM), fatty-acid free BSA (3 mg ml^-1^), KCN (0.3 mM) and NADH (0.1 mM). A blank rate was measured for 2 minutes before ubiquinone_1_ (CoQ_1_) (60 μM) was added to the cuvette to start the reaction and a decrease in absorbance was monitored for 3 minutes. Rotenone (10 μM) was then added and the rate was monitored for a further 2 minutes. Specific complex I activity was taken as the rotenone-sensitive activity determined by subtracting the rotenone-resistant activity from the total activity.

#### Succinate dehydrogenase (Complex II) activity assay

The activity of succinate dehydrogenase (Complex II) was determined by monitoring the reduction of 2,6-dichlorophenolindophenol (DCPIP) at 600 nm, using an assay based on the method of Hatefi [30]. Homogenate samples (approximately 20 μg) were incubated at 30°C in a 1 ml cuvette of assay buffer consisting of potassium phosphate pH 7.5 (25 mM), succinic acid (20 mM), fatty-acid free BSA (1 mg ml^-1^), KCN (0.3 mM) and 2,6-dichlorophenolindophenol (DCPIP) (0.002%) for 8 minutes. A blank rate was then measured for 2 minutes before decylubiquinone (50 μM) was added to the cuvette to start the reaction and a decrease in absorbance was monitored for 3 minutes. Malonate (10 mM) was subsequently added to inhibit the enzymatic reaction and the absorbance was monitored for an additional 3 minutes. Specific complex II activity was determined by subtracting the malonate insensitive activity from the total activity.

#### Decylubiquinol cytochrome c oxidoreductase (Complex III) activity assay

The activity of decylubiquinol cytochrome *c* oxidoreductase (Complex III) was determined by monitoring the reduction of cytochrome *c* at 550 nm, by a method adapted from that described by Ragan *et al*. [29]. Homogenate samples (approximately 20 μg) were incubated in a 1 ml cuvette of assay buffer consisting of the following reaction components: potassium phosphate pH 7.5 (25 mM), oxidised cytochrome *c* (75 μM), EDTA Solution, pH 7.5 (0.1 mM), KCN (0.3 mM) and Tween-20 (0.025%). A blank rate was measured for 2 minutes before decylubiquinol solution (0.1 mM) was then added to initiate the reaction and an increase in absorbance was monitored for 2 minutes. Decylubiquinol solution was freshly prepared prior to use. Antimycin A (10 μg ml^-1^) was subsequently added and monitored for a further 2 minutes. Specific complex III activity was taken as the antimycin A-sensitive activity determined by subtracting the antimycin A-resistant activity from the total activity.

#### Cytochrome c oxidase (Complex IV) activity assay

The activity of cytochrome *c* oxidase (Complex IV) was determined by monitoring the oxidation of reduced cytochrome *c* at 550 nm, using an assay based on the method described by Wharton and Tzagoloff [31]. Reduced cytochrome *c* (1 mM) was freshly prepared by dissolving oxidised cytochrome *c* in 20 mM potassium phosphate buffer (pH 7.0) and reducing the solution with sodium dithionite A 1 ml cuvette containing the assay mixture consisting of potassium phosphate pH 7.0 (50 mM) and reduced cytochrome *c* (60 μM) was placed in the spectrophotometer and a blank rate was measured for 2 minutes. Homogenate (approximately 10 μg) was then added to the cuvette to initiate the reaction and a decrease in absorbance was monitored for 2 minutes. The ‘apparent linear’ rate was measured over the same time-frame for each measurement. KCN (0.3 mM) was subsequently added to inhibit the enzymatic reaction and the absorbance was monitored for a further 2 minutes. Specific complex IV activity was determined by subtracting the KCN insensitive activity from the total activity.

#### NADH cytochrome c oxidoreductase (Complex I+III) activity assay

The activity of NADH cytochrome *c* oxidoreductase (Complex I+III) was determined by monitoring the reduction of cytochrome *c* at 550 nm, as per the method described by Powers *et al*. [32]. Homogenate samples (approximately 20 μg) were incubated in dH_2_O in a 1 ml cuvette in order to allow osmotic shock to occur. After two minutes incubation the reaction components were added: potassium phosphate pH 7.5 (50 mM), oxidised cytochrome *c* (50 μM), KCN (0.3 mM), and fatty-acid free BSA (1 mg ml^-1^) and a blank rate was measured for 2 minutes. NADH (0.2 mM) was then added to initiate the reaction and an increase in absorbance was monitored for 3 minutes. Rotenone (10 μM) was then added and the rate was monitored for a further 2 minutes. Complex I+III combined specific activity was taken as the rotenone-sensitive activity determined by subtracting the rotenone-resistant activity from the total activity. In a separate experiment repeated in the same manner, ubiquinone_1_ (coenzyme Q_1_) (CoQ_1_) at a final concentration of 100 μM was added after the initiation and monitoring of the reaction with NADH and prior to the addition of rotenone. The absorbance was monitored for 3 minutes before the addition of rotenone as previous.

#### Succinate cytochrome c reductase (Complex II+III) activity assay

The activity of succinate cytochrome *c* reductase (Complex II+III) was determined by monitoring the reduction of cytochrome *c* at 550 nm, using an assay based on the method of King [33]. Homogenate samples (approximately 20 μg) were incubated at 30°C in a 1 ml cuvette of assay buffer consisting of potassium phosphate pH 7.5 (20 mM), succinic acid (10 mM) and KCN (0.3 mM) for 8 minutes. A blank rate was then measured for 2 minutes before oxidised cytochrome *c* (50 μM) was added to the cuvette to initiate the reaction and an increase in absorbance was monitored for 3 minutes. Malonate (10 mM) was subsequently added to inhibit the enzymatic reaction and the absorbance was monitored for an additional 2 minutes. Complex II+III combined specific activity was determined by subtracting the malonate insensitive activity from the total activity. In a separate experiment repeated in the same manner, ubiquinone_1_ (CoQ_1_) at a final concentration of 100 μM was added after the initiation and monitoring of the reaction with oxidised cytochrome *c* and prior to the addition of malonate. The absorbance was monitored for 3 minutes before the addition of malonate as previous.

#### Specific enzyme activity and molar extinction coefficients

Specific enzyme activities were expressed as nanomoles per minute per milligram of muscle protein. The molar extinction coefficients used were 13,600 L mol^-1^ cm^-1^ for citrate synthase at 412 nm, 6,200 L mol^-1^ cm^-1^ for Complex I at 340 nm, 19,100 L mol^-1^ cm^-1^ for Complex II at 600 nm, 18,500 L mol^-1^ cm^-1^ for Complex III at 550 nm, 18,500 L mol^-1^ cm^-1^ for Complex IV at 550 nm, 18,500 L mol^-1^ cm^-1^ for Complex I+III at 550 nm and 18,500 L mol^-1^ cm^-1^ for Complex II+III at 550 nm. Mitochondrial complex activities were expressed as a ratio to citrate synthase activity, in order to account for the mitochondrial enrichment of the skeletal muscle homogenates.

### Quantitative PCR–mitochondrial DNA:nuclear DNA ratio

The cytochrome *c* oxidase subunit 1 (*CO1*) gene of the mitochondrial DNA (mtDNA) and the NADH:ubiquinone oxidoreductase core subunit V1 (*NDUFV1*) gene of the nuclear DNA (nDNA) were used to measure the relative mtDNA:nDNA ratio. The primers were as follows; *CO1*:5-TCC TAG CAG CAG GCA TAA C-3 (forward), 5-GGG TGT CCG AAG AAT CAG AAT-3 (reverse), *NDUFV1*: 5-CTT CCC CAC TGG CCT CAA-3 (forward), 5-TCC AAG GAA AGA GCA AAG GC-3 (reverse). QPCR was performed using SYBR green qPCR mix (Life Technologies) on an Applied Biosystems 7500 Fast Real-Time PCR System. Equal amounts of DNA were used for each reaction and combined with forward primer (0.5 μM), reverse primer (0.5 μM), SYBR green master mix and nuclease free water in a 96 well reaction plate (Applied Biosystems). PCR conditions were the same for all primers sets: initial denaturation at 95°C for 10 minutes, followed by 40 cycles of 95°C for 10 seconds and 60°C for 30 seconds. All reactions were run in triplicate. SDS 1.9.1 software (Applied Biosystems) was used to analyse the amplification curves and these curves were used to determine the relative mtDNA:nDNA ratio in each sample. Careful attention was paid to avoid PCR contamination and no false-positives were observed in negative controls. Visualisation of qPCR amplification products on a 1.5% agarose gel was used to confirm the specificity of the reaction (*i*.*e*. free of non-specific bands).

### Gene expression

Total RNA was isolated from skeletal muscle tissue samples using Qiazol reagent (Qiagen) and homogenisation using 1.5 mm stainless steel beads in a Tissue Lyser II machine (Qiagen) before being isolated and purified using RNeasy Plus Universal kit (Qiagen) as per the manufacturer’s instructions. Equal amounts of RNA were reverse transcribed into cDNA using the High-Capacity cDNA Reverse Transcription Kit (Applied Biosystems) as per manufacturer’s instructions. Reverse transcription was performed by heating the reaction mix at 25°C for 10 minutes followed by 37°C for 120 minutes and 85°C for 5 minutes. The resulting cDNA was then diluted with nuclease free water and used for real-time qPCR. Specific primers (S1 Table) were designed with the aid of Primer3Plus [34], spanning exon:exon junctions where possible. Primers for the myosin heavy chain isoform genes were designed for regions previously described in the corresponding ovine genes [35]. All primers were commercially synthesised (Eurofins Genomics). Sequence homology to other genomic regions was assessed using the National Center for Biotechnology Information BLAST function [36]. Hypoxanthine guanine phosphoribosyl transferase (*HPRT*) mRNA expression was used as an internal normalization control for each sample, as it was reported to be the most stably expressed gene when a panel of potential reference genes were screened in horses [37]. Careful attention was paid to avoid PCR contamination and no false-positives were observed in negative controls. Biosystems 7500 Fast Real-Time PCR System and SYBR green reagents were used to measure mRNA in equine skeletal muscle tissue. PCR conditions were as follows: initial denaturation at 95°C for 10 minutes, followed by 40 cycles of 95°C for 10 seconds and 58°C (*HPRT*, *MYH7*, *MYH2*, *MYH1*) or 60°C (*PDSS1*, *PDSS2*, *COQ2*, *COQ3*, *COQ4*, *COQ5*, *COQ6*, *COQ7*, *ADCK3*, *ADCK4* and *COQ9*) for 30 seconds. All reactions were run in at least duplicate. SDS 1.9.1 software (Applied Biosystems) was used to analyse the amplification curves and these curves were used to determine the relative mRNA expression of each gene. The expression of each gene was normalised to the expression of *HPRT* using the ΔΔCt method. Myosin heavy chain (MHC) isoform gene expression (*MYH7*, *MYH2* and *MYH1*) was subsequently expressed as a percentage of total MHC gene expression.

### SDS-PAGE and immunoblotting

Proteins were resolved by sodium dodecyl sulphate-polyacrylamide gel electrophoresis (SDS-PAGE) as per the method of Laemmli [38] and transferred to polyvinylidene difluoride (PVDF) membranes (Immobolin-P^SQ^; Sigma-Aldrich) using a semi-dry transfer system (Hoefer Inc.). Membranes were blocked by incubation in TBS-Tween (TBS supplemented with 0.1% (v/v) Tween) (TBST) supplemented with 5% (w/v) non-fat dry milk powder for 1 hour at room temperature. Blots were then incubated in primary antibody (mouse monoclonal anti-MHC slow (M8421 Sigma-Aldrich) at 1:1000; mouse monoclonal anti-MHC fast (M4276 Sigma-Aldrich) at 1:1000; rabbit polyclonal anti-COQ4 (ab126295 AbCam) at 1:1000; rabbit polyclonal anti-ADCK3 (ab124237 AbCam) at 1:1000; mouse polyclonal anti-COQ3 (ab88561 AbCam) at 1:1000; and mouse monoclonal anti-GAPDH (CB1001 Calbiochem) at 1:2000) diluted in TBST supplemented with 5% (w/v) non-fat dry milk powder overnight at 4°C. Following primary antibody incubation, the membranes were washed and subsequently incubated in the appropriate horseradish peroxidase (HRP) conjugated secondary antibody (Rabbit 1:5000 or Mouse 1:5000 (Promega)) diluted in TBST supplemented with 5% (w/v) non-fat dry milk powder, for 1 hour at room temperature. Blots were developed using an enhanced chemiluminescence (ECL) detection system (Millipore Immobilon ECL Substrate) for detecting horseradish peroxidase labelled antibody, by means of the HRP catalysed oxidation of luminol under alkaline conditions and the results were visualised by ChemiDoc (Bio-Rad) computerised system and Image Lab software. Densitometry analysis was performed using Image Lab Software Analysis function and/or Image J software [39].

### Statistical analysis

Statistical analyses were performed using the computer-based mathematical package Graph Pad Prism software. Power analyses were performed using PS-Power and Sample Size Calculations software. An initial sample set of n = 4 horses per *MSTN* genotype was used to estimate the sample size required for the study. The sample sizes required ranged from 6 to 16 depending on the assay data tested, with a value of 0.05 for significance, power of 0.8 and the assumption of equal group numbers. The total population was an unbalanced 82 samples, therefore this power calculation was used as an estimate. All results are expressed as mean ± SEM unless otherwise indicated. Mean values were compared using a one-way ANOVA (means (fixed) model: $y_{ij}=\mu_{j}+\varepsilon_{ij}$) with a Bonferroni multiple comparison post-test with 95% confidence intervals, as appropriate. A p-value of ≤ 0.05 indicated significance, corresponding to the applied confidence interval of 95%.

## Results

### No confounding effects of age, conformation or exercise phenotypes

The two *MSTN* polymorphisms (g.66493737C>T and SINE insertion) were in complete concordance in the study population. Therefore, the effects of the two polymorphisms were considered together and the genotypes were denoted CC/II (homozygous C allele / insertion allele), CT/IN (heterozygous) and TT/NN (homozygous T allele / no insertion allele). The genotype distributions were CC/II n = 37, CT/IN n = 34 and TT/NN n = 11.

There was no confounding effect of age, conformation or exercise phenotypes on the measured mitochondrial phenotypes since there were no significant differences among genotypes. The subjects were aged 21 ± 3 months at the time of sampling and the genotype and sex distributions were CC/II n = 37 (15 male, 22 female), CT/IN n = 34 (15 male, 19 female) and TT/NN n = 11 (1 male, 10 female). Due to the presence of only one male in the TT/NN group, the effects of sex on the mitochondrial phenotypes could not be considered in the analysis. Body weight and wither height were measured within 60 days from the date of biopsy for as many of the study cohort as possible. While there was a significant difference in body weight between the CC/II (n = 24) and CT/IN (n = 17) horses (p < 0.05) (S1 Fig) there was no significant association between body weight/wither height and genotype (CC/II: n = 24, CT/IN: n = 17 and TT/NN; n = 6) (S1 Fig). The amount of submaximal training performed by each horse was evaluated to ensure no confounding effects of exercise. While the amount of submaximal training varied among individuals there was no significant association between submaximal training (measured in days from ‘breaking’ to biopsy and days from first canter [moderate intensity exercise] to biopsy), and genotype (S2 Fig).

### Mitochondrial content variation among genotypes

**Fig 1:**
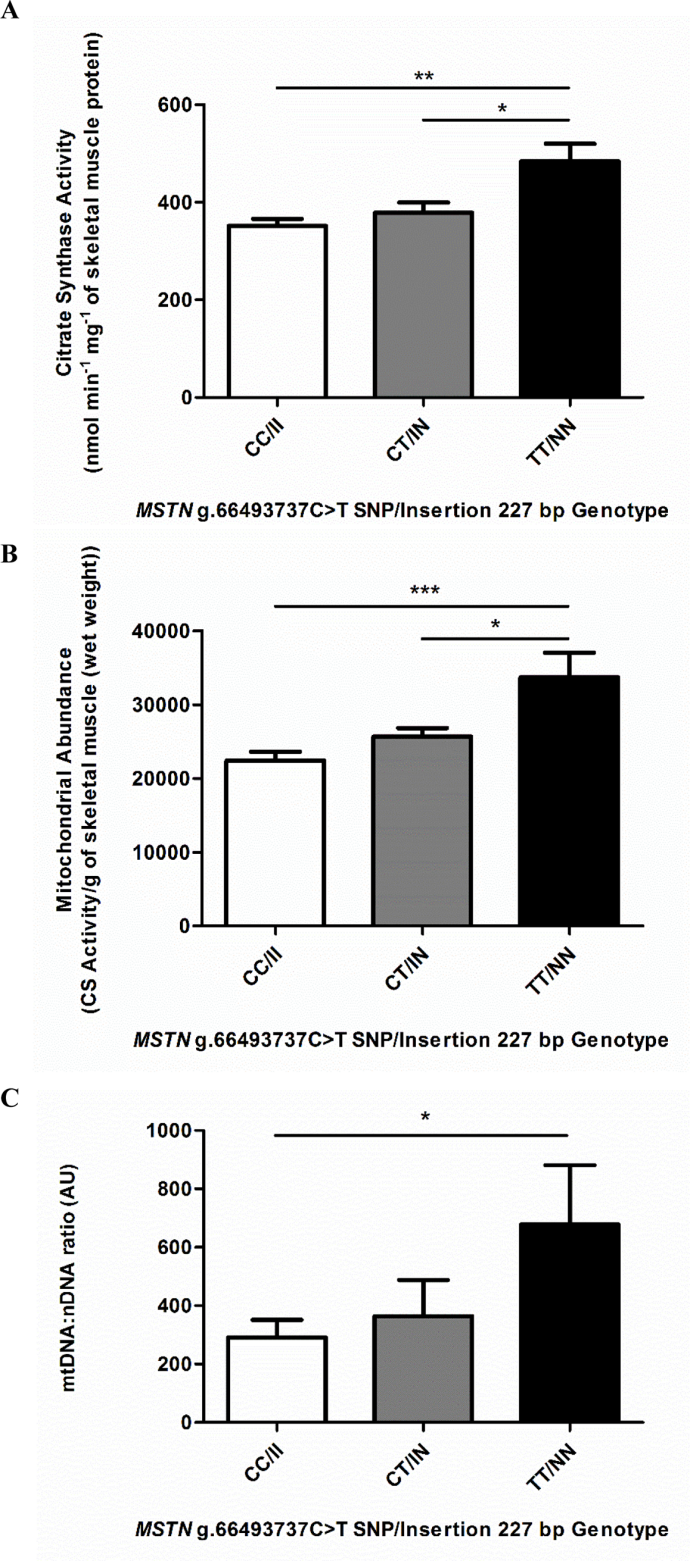
Skeletal muscle mitochondrial content of *MSTN* genotype (CC/II, CT/IN, TT/NN) horses. Mitochondrial abundance determined by the activity of citrate synthase (CS) measured spectrophotometrically, expressed (A) as nmol/min/mg of muscle protein and (B) as nmol/min/g of skeletal muscle (wet weight), CC/II: n = 37, CT/IN: n = 32 and TT/NN: n = 11, performed in at least duplicate; and (C) mtDNA:nDNA ratio measured by qPCR, CC/II: n = 19, CT/IN: n = 17 and TT/NN: n = 8, performed in at least triplicate. Results presented with mean ± SEM, p-values where shown indicate significance as measured by a one-way ANOVA with a Bonferroni multiple comparison post-test, * = p ≤ 0.05, ** = p ≤ 0.01, *** = p ≤ 0.001.

The activity of the mitochondrial citrate synthase (CS; expressed as the mean ± SEM (n) nmol/min/mg of muscle protein), used to determine mitochondrial abundance in skeletal muscle, was significantly (p ≤ 0.01) lower in CC/II horses (CC/II: 351.6 ± 14.8 (37)) compared to TT/NN horses (TT/NN: 484.3 ± 35.7 (11)) (Fig 1A). Heterozygotes had significantly (p ≤ 0.05) lower CS activity compared to TT/NN horses (CT/IN: 378.9 ± 20.7 (32)) but no difference was observed between CC/II horses and CT/IN horses. Mitochondrial abundance, expressed as CS activity/g wet weight of skeletal muscle was also significantly (CC/II versus TT/NN, p ≤ 0.001; CT/IN versus TT/NN, p ≤ 0.05) lower in the presence of the C / I allele (CC/II: 22410 ± 1235 (37); CT/IN: 25630 ± 1168 (32); TT/NN: 33680 ± 3371 (11)) (Fig 1B) but no difference was observed between CC/II and CT/IN horses. In an independent assay, the mitochondrial DNA:nuclear DNA ratio (Fig 1C) confirmed the lower mitochondrial content of CC/II horses compared to TT/NN horses (p ≤ 0.05) but not between CC/II and CT/II or between CT/IN and TT/NN horses.

### Muscle fibre type variation among genotypes

**Fig 2:**
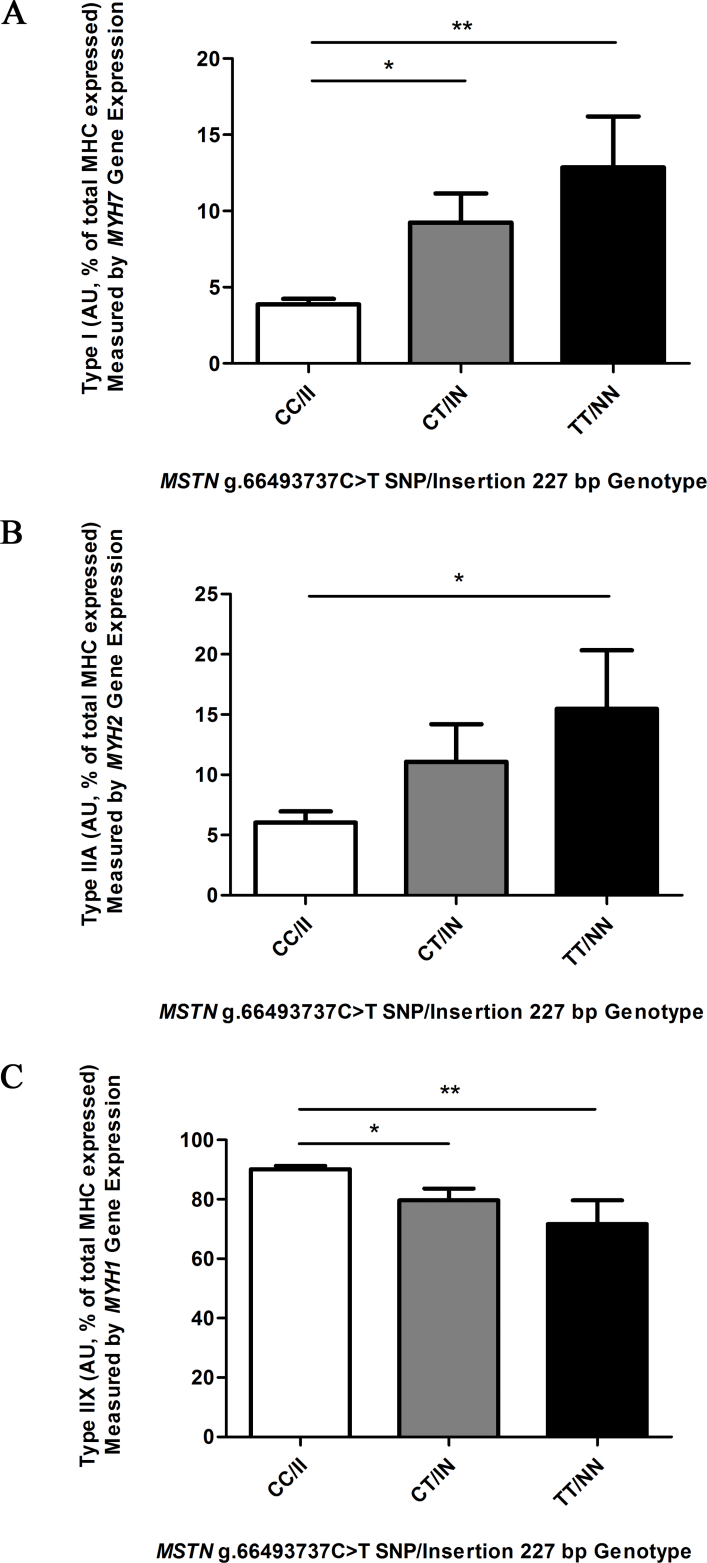
Skeletal muscle fibre type proportions among *MSTN* genotype (CC/II, CT/IN, TT/NN) horses. qPCR was used to measure gene expression levels of three genes: *MYH7* (A), *MYH2* (B) and *MYH1* (C) inferring MHC isoforms and interpreted as Type I, Type IIA and Type IIX fibres, respectively among CC/II: n = 36, CT/IN: n = 34 and TT/NN: n = 11, performed in at least duplicate. Gene expression was normalised to *HPRT* using the ΔΔCt method and expressed as a percentage of total MHC gene expression. Results presented with mean ± SEM, p-values where shown indicate significance as measured by a one-way ANOVA with a Bonferroni multiple comparison post-test, * = p ≤ 0.05, ** = p ≤ 0.01, *** = p ≤ 0.001.

**Fig 3:**
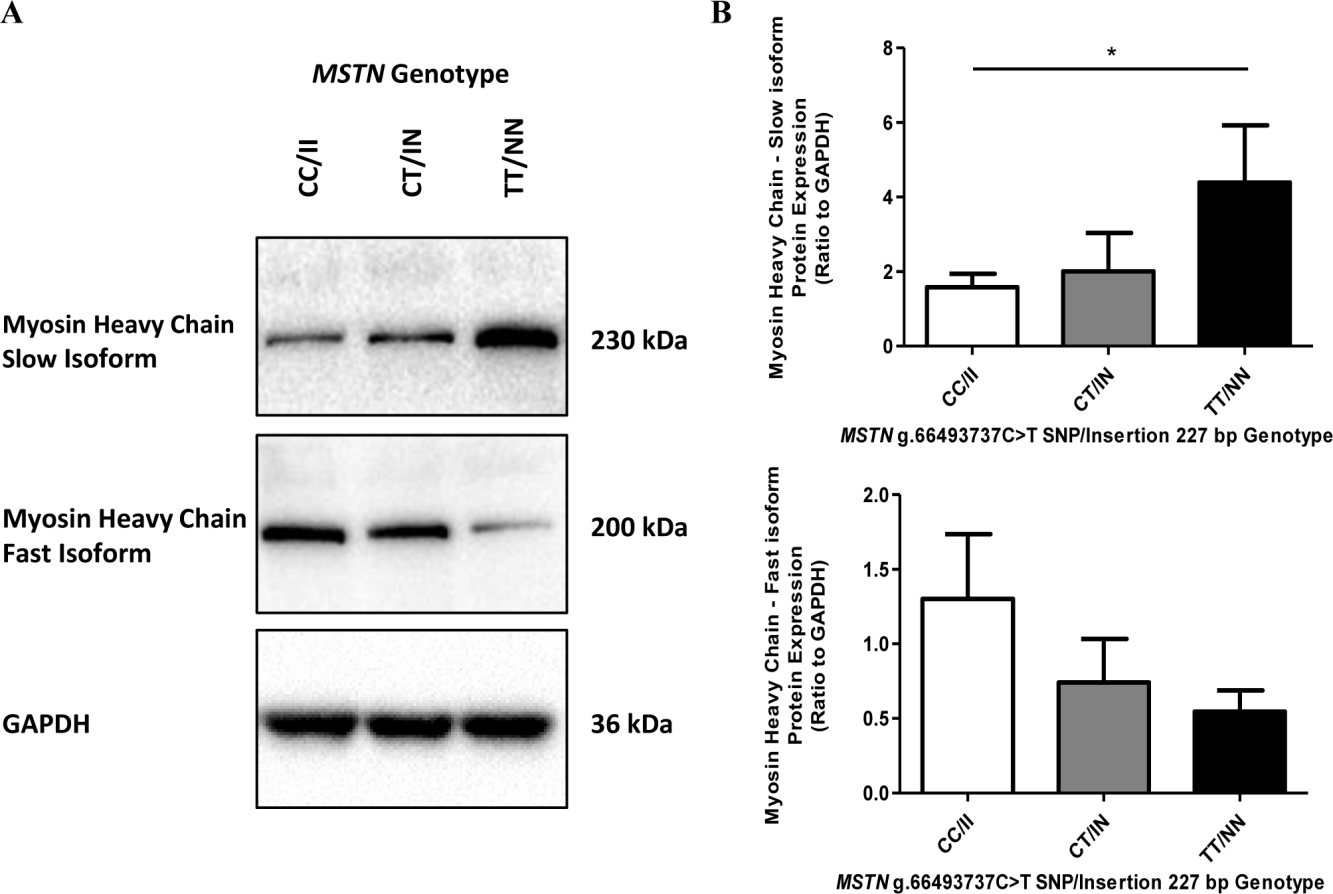
Myosin heavy chain protein expression in *MSTN* genotype (CC/II, CT/IN, TT/NN) horses. (A) Myosin heavy chain slow isoform and myosin heavy chain fast isoform protein levels in muscle protein lysates (CC/II: n = 6, CT/IN: n = 6 and TT/NN: n = 6) measured by immunoblot analysis. A representative experiment out of the six performed is shown for each protein. (B) Densitometry results are shown for the slow isoform and the fast isoform, presented with mean ± SEM, p-values where shown indicate significance as measured by a one-way ANOVA with a Bonferroni multiple comparison post-test, * = p ≤ 0.05, ** = p ≤ 0.01, *** = p ≤ 0.001.

Considering the previously observed differences in muscle fibre proportions associated with *MSTN* genotype in a small set of Thoroughbreds (n = 25) [23], we analysed a larger set of Thoroughbred horses (n = 81; CC/II n = 36, CT/IN n = 34 and TT/NN n = 11), to measure the fibre composition of the skeletal muscle in the untrained state and confirm the previous observed relationship between muscle fibre composition and *MSTN* genotype. Fibre proportions were indirectly determined by measuring mRNA of genes producing myosin heavy chain isoforms that differ among the three fibre types, and were expressed as a percentage of the total. The *MYH7* gene produces myosin heavy chain (MHC) isoform I protein which is characteristic of type I fibres, the *MYH2* gene produces MHC isoform IIA which is characteristic of type IIA fibres and the *MYH1* gene produces MHC isoform IIX which is characteristic of type IIX fibres. Significant muscle fibre proportion differences were observed among the genotypes. CC/II horses had a significantly lower proportion of type I fibres compared to CT/IN horses (p ≤ 0.05) and TT/NN (p ≤ 0.01) horses (Fig 2A). A similar profile was observed for type IIA fibres, with the TT/NN horses having a significantly (p ≤ 0.05) higher proportion compared to CC/II horses (Fig 2B). In contrast, TT/NN (p ≤ 0.01) and CT/NN horses had significantly (p ≤ 0.05) fewer type IIX fibres compared to CC/II horses (Fig 2C). A subset of the horses (n = 6 per genotype) was also analysed by immunoblot for the expression of slow and fast isoforms of the myosin heavy chain protein. Densitometry analysis indicated that the TT/NN horses had significantly more of the slow isoform myosin heavy chain protein than the CC/II genotype horses (p ≤ 0.05) (Fig 3). By comparison, the CC/II horses appeared to have a greater amount of the fast isoform of the myosin heavy chain protein than the TT/NN genotype horses, though this was not statistically significant considering the densitometry analysis (p = 0.15). The immunoblot data reflects the qPCR data indicating a significant association between *MSTN* genotype and muscle fibre composition in untrained Thoroughbred skeletal muscle.

### Mitochondrial bioenergetic variation among genotypes

**Fig 4:**
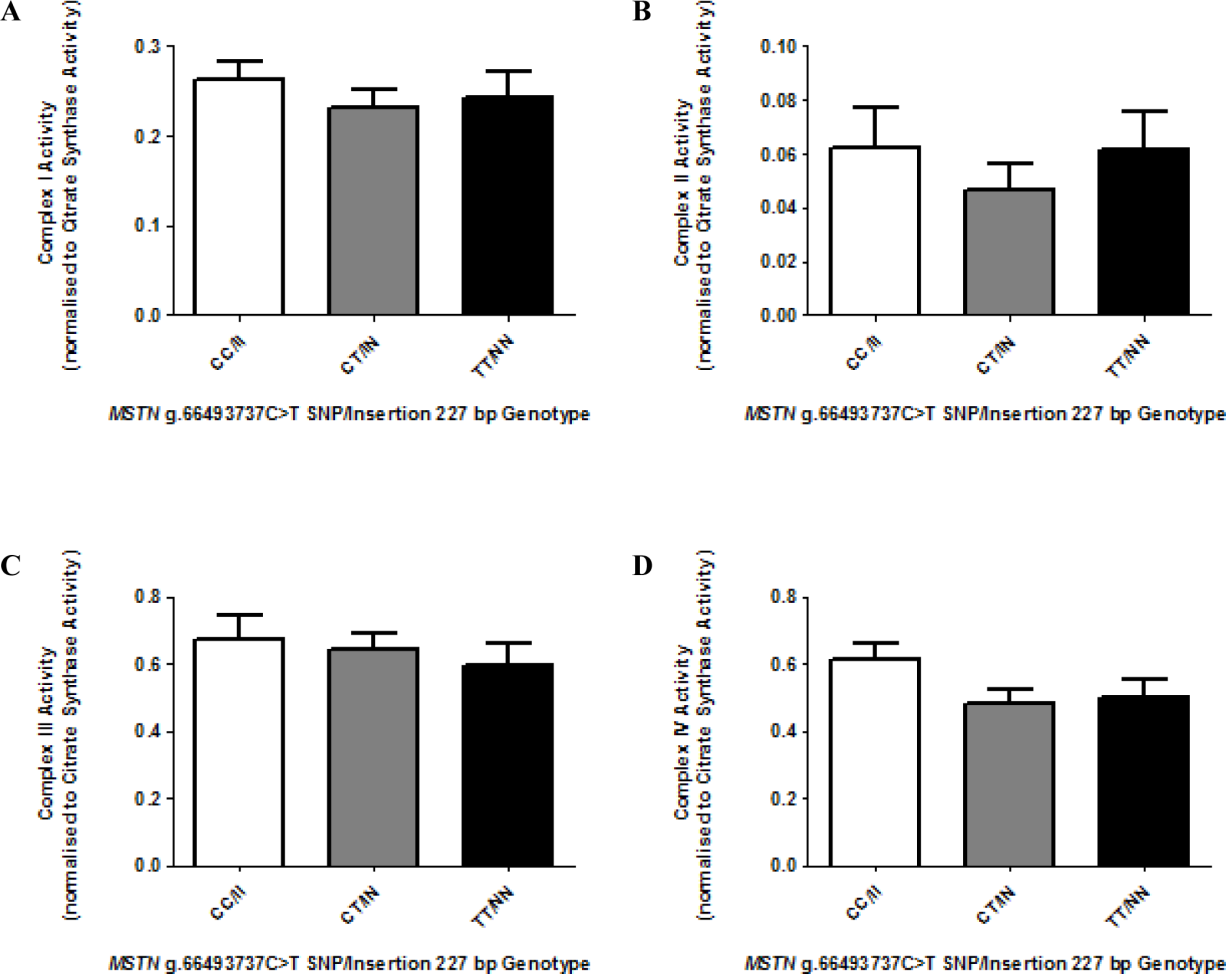
Individual electron transport chain complex enzyme activities among *MSTN* genotype (CC/II, CT/IN, TT/NN) horses. NADH-ubiquinone oxidoreductase (Complex I), Succinate dehydrogenase (Complex II), Decylubiquinol cytochrome c oxidoreductase (Complex III) and Cytochrome c oxidase (Complex IV) (A-D, respectively) activities were measured spectrophotometrically in whole skeletal muscle homogenates (CC/II: n = 20, CT/IN: n = 15 and TT/NN: n = 8), performed in at least duplicate. Results presented with mean ± SEM, p-values where shown indicate significance as measured by a one-way ANOVA with a Bonferroni multiple comparison post-test.

The activities of individual mitochondrial electron transport chain complexes, including NADH:ubiquinone oxidoreductase (complex I) (Fig 4A), succinate dehydrogenase (complex II) (Fig 4B), ubiquinol-cytochrome c oxidoreductase (complex III) (Fig 4C) and cytochrome c oxidase (complex IV) (Fig 4D) were measured, normalized to mitochondrial abundance (CS activity) to determine the specific complex activity per unit mass of mitochondria and compared among genotypes. ATP synthase (Complex V) activity was not measured as its reliability in frozen tissue samples is questionable due to high oligomycin-resistant activities [40, 41]. There was no observable difference among genotypes for any of the individual mitochondrial complex (I-IV) activities (p > 0.05) (Fig 4, S2 Table).

**Fig 5:**
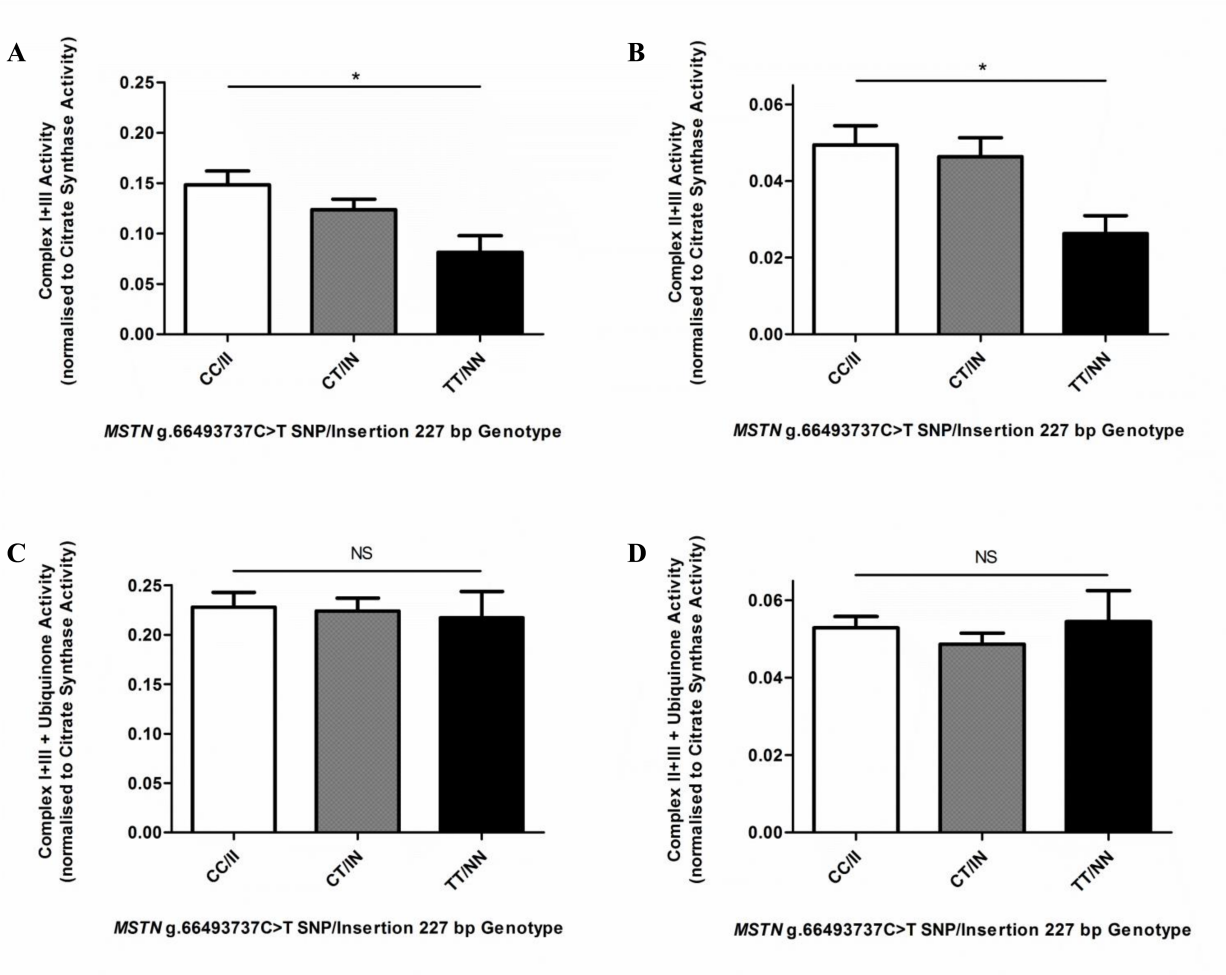
CoQ (ubiquinone) activity, measured by combined complex activities, among *MSTN* genotype (CC/II, CT/IN, TT/NN) horses. NADH cytochrome c oxidoreductase (Complex I + III) (A and C) and Succinate cytochrome c reductase (Complex II + III) (B and D) activities were measured spectrophotometrically on whole skeletal muscle homogenates (A) CC/II: n = 29, CT/IN: n = 28 and TT/NN: n = 7, (B) CC/II: n = 37, CT/IN: n = 32 and TT/NN: n = 11, (C) CC/II: n = 29, CT/IN: n = 28 and TT/NN: n = 7, and (D) CC/II: n = 33, CT/IN: n = 28 and TT/NN: n = 7, all performed in at least duplicate. CI+III (A) and CII+III (B) activities are an indirect measure of CoQ (ubiquinone). CI+III (C) and CII+III (D) activities were measured in the presence of supplementary CoQ_1_ (Ubiquinone_1_), demonstrating the relative complex activity deficiency is due to lower levels of endogenous CoQ. Results presented with mean ± SEM, p-values where shown indicate significance as measured by a one-way ANOVA with a Bonferroni multiple comparison post-test, * = p ≤ 0.05, ** = p ≤ 0.01, *** = p ≤ 0.001.

The combined enzyme activities of complex I+III and complex II+III were measured, normalized to mitochondrial abundance (CS activity) to determine the specific complex activity per unit mass of mitochondria and compared among genotypes. There were significantly lower levels of both combined complex I+III (Fig 5A) and complex II+III (Fig 5B) activities in TT/NN horses compared to CC/II horses (p ≤ 0.05) (CI+III = CC/II: 0.1483 ± 0.0137 (n = 29); CT/IN: 0.1237 ± 0.0104 (n = 28) TT/NN: 0.0812 ± 0.0166 (n = 7); CII+III = CC/II: 0.04942 ± 0.00500 (n = 37) CT/IN: 0.04633 ± 0.00500 (n = 32) TT/NN: 0.02630 ± 0.00465 (n = 11)). TT/NN horses had approximately half the combined complex activities of CC/II horses (p ≤ 0.05).

Considering that the individual activities of complex I, II and III appeared to be unaffected by genotype the differences in the activities of the combined complexes suggested there may be lower levels of CoQ in the mitochondria of skeletal muscle tissue of TT/NN horses compared to CC/II. CoQ_10_ (ubiquinone (oxidized from) or ubiquinol (reduced form)) acts as a mobile redox carrier linking complex I and II with complex III in the electron transport chain of mitochondria. The assays measuring the combined enzyme activities of complex I+III and complex II+III may be used as an indirect measure of CoQ levels in the mitochondria in tissue samples. Therefore, to test the hypothesis that the combined complex activities were lower in TT/NN horses as a result of lower endogenous CoQ availability we conducted an ‘add-back’ experiment by adding CoQ_1_ (or ubiquinone_1_) to the complex I+III and complex II+III assays. The addition of ubiquinone restored the activity of complex I+III and complex II+III in the TT/NN horses to similar levels measured in the CC/II horses, indicating the difference in complex activity was due to a relative deficiency in endogenous CoQ in TT/NN horses (Fig 5C and 5D).

**Fig 6:**
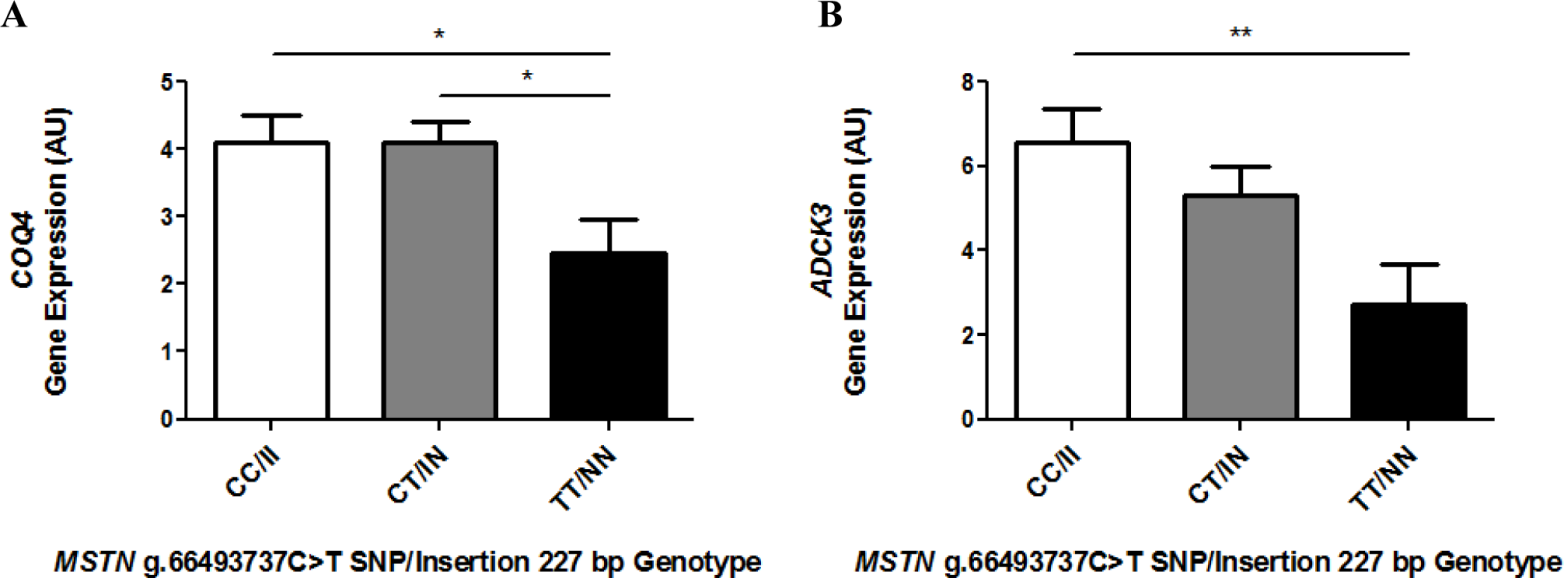
Expression of CoQ biosynthesis genes in *MSTN* genotype (CC/II, CT/IN, TT/NN) horses. *COQ4* (A) and *ADCK3* (B) gene expression was determined for CC/II: n = 36, CT/IN: n = 34 and TT/NN: n = 11, performed in at least duplicate. Gene expression was normalised to *HPRT* using the ΔΔCt method. Results presented with mean ± SEM, p-values where shown indicate significance as measured by a one-way ANOVA with a Bonferroni multiple comparison post-test, * = p ≤ 0.05, ** = p ≤ 0.01, *** = p ≤ 0.001.

To further explore the apparent relative CoQ deficiency in TT/NN skeletal muscle mitochondria, the expression of 11 genes (*PDSS1*, *PDSS2*, *COQ2*, *COQ3*, *COQ4*, *COQ5*, *COQ6*, *COQ7*, *ADCK3*, *ADCK4* and *COQ9*) encoding enzymes and proteins involved in the biosynthesis of CoQ was analysed. The relative expression of two genes *COQ4* and *ADCK3* was significantly (*COQ4*, p ≤ 0.05; *ADCK3*, p ≤ 0.01, Fig 6) lower in skeletal muscle of TT/NN horses compared to CC/II horses. Heterozygous CT/IN animals also had significantly higher *COQ4* expression than TT/NN horses (p ≤ 0.05). There was no significant association with *MSTN* genotype for the other CoQ pathway genes, though there was an observable trend towards lower expression in TT/NN horses compared to CC/II horses (S3 Fig). Considering the observed differences in transcripts for *COQ4* and *ADCK3*, we examined whether the differences in transcripts manifested themselves as differences in protein expression in the skeletal muscle. Expression levels of COQ3, COQ4 and ADCK3 proteins were measured by immunoblot analysis in a subset (n = 6 per genotype) of samples (S4 Fig). No significant association (p > 0.05) was observed between *MSTN* genotype and the expression of COQ4, ADCK3 and COQ3 proteins.

## Discussion

*MSTN* polymorphisms (g.66493737C>T and SINE insertion) in Thoroughbreds are associated with optimum race distance [17, 18] and muscle fibre composition [22]. We hypothesised that *MSTN* variation effects mitochondrial abundance and bioenergetics, which subsequently manifest in the system-wide phenotypes relevant to racecourse performance that have previously been reported. In this study we observed that variation in *MSTN* genotype is associated with mitochondrial abundance and the presence of the C allele / I allele corresponded with a lower proportion of skeletal muscle mitochondria. For context, we measured variation in skeletal muscle fibre proportions and found the T allele / N allele corresponded to an increased proportion of type I and type IIA fibres in combination with a decreased proportion of type IIX fibres. These data are consistent with previously reported histochemistry findings [23]. Petersen *et al*. [23] have provided evidence from analyses of muscle fibre composition in the Belgian horse breed, which does not have the SINE insertion polymorphism, that the SINE insertion and not the SNP g.66493737C>T most likely has the predominant effect on the variation in muscle fibre composition. In the present study the SNP and SINE insertion are in complete concordance, therefore the association with muscle fibre proportions was observed for both polymorphisms.

Type I skeletal muscle fibres are more oxidative, contain more mitochondria and lend to greater endurance capacity than glycolytic type IIX skeletal muscle fibres. Type IIA fibres are intermediate in their oxidative and glycolytic capacities and similar to type I fibres, have a high mitochondrial content [42–44]. Considering this, it is likely that the observed association between genotype and mitochondrial abundance is a consequence of the muscle fibre composition variation and not an independent effect. Since the fibre composition of skeletal muscle determines the contractile pattern of the muscle and hence the potential for physical performance [45] this likely explains the observation of the increased stamina performance of TT/NN horses and the greater suitability for sprint racing of CC/II horses [16, 17, 19, 20].

Endurance training is known to increase mitochondrial abundance in skeletal muscle and results in a shift in fibre proportions towards a more oxidative phenotype with an increase in type I fibres [43, 46]. Similar alterations are expected to occur in this cohort after a period of training, but whether those changes would be influenced by *MSTN* genotype would need to be examined.

While there does not, therefore, appear to be a direct effect of *MSTN* variation on mitochondrial abundance, we examined whether *MSTN* variation influenced the energetic phenotype of the mitochondria by measuring mitochondrial electron transport chain complex activity. Since there was no association with *MSTN* genotype and the individual complexes I, II, III or IV, this supports the assertion that the variation in mitochondrial abundance is likely an indirect effect of the association with muscle fibre proportions rather than a direct effect on the mitochondria.

Notwithstanding this, we observed significant variation in combined complex I+III and complex II+III activities associated with *MSTN* genotype. CoQ acts as a mobile redox carrier linking complexes I and II with complex III. Considering the individual electron transport chain complex activities were not influenced by *MSTN* genotype, the variation in the combined activity assays (CI+III and CII+III) suggested lower CoQ concentrations in the skeletal muscle mitochondria of TT/NN horses; however, a definitive measure of CoQ_10_ by high-performance liquid chromatography was not feasible here. Mitochondrial content was used to normalise the complex activity measurements, therefore it may be interpreted that the differences in CI+III and CII+III activities are independent of the association of *MSTN* genotype with mitochondrial abundance. The ‘add back’ of ubiquinone to the assays removed any difference in genotype variation, suggesting that endogenous CoQ production in TT/NN horses is lower than in CT/IN and CC/NN horses. In addition, significant gene expression variation in two key CoQ synthesis pathway genes (*COQ4* and *ADCK3*) was associated with *MSTN* genotype. *COQ4* is thought to encode a zinc binding protein which provides a structural centre holding the enzymes of CoQ biosynthesis together in a complex. A decrease in the expression of *COQ4* could reduce the stability of the complex and hence reduce the efficiency of CoQ biosynthesis. *ADCK3* is thought to encode a protein which may act in a chaperone role in the biosynthetic process and so decreased *ADCK3* would also have an impact on the efficiency of CoQ biosynthesis. Previously, mutations in *ADCK3* and *COQ4* have been observed to result in decreased CoQ levels in skeletal muscle [47–49].

However, protein levels, as measured by immunoblotting were not different among the genotypes suggesting that the transcript level is decreased without a coinciding decrease in protein level. Although seemingly unusual, it is frequent to observe protein levels which do not reflect the transcriptional expression of the encoding gene [50]. This lack of correlation is mainly due to the extent of the regulatory control of gene expression and protein synthesis. Studies have found that in some systems the correlation between the relative mRNA and protein abundances can be as low as 40%, at steady state [51, 52]. The remaining variation is thought to be a result of post-transcriptional regulation and experimental measurement noise. The processes of transcription, translation and protein degradation are often coupled and thus regulate one another in a feedback loop mechanism, which may result in a lack of correlation between mRNA and protein abundance [53, 54]; however, a full interpretation of the variation has yet to be made. Here, we have observed decreased CoQ levels, as measured indirectly by combined complex activities along with a corresponding decreased expression of two CoQ biosynthesis genes. These associations were observed without a corresponding protein variation which may mean that the decreased gene expression is not resulting in any protein variation due to the aforementioned lack of correlation explanations. Although without an association with the functional protein one may not conclude a functional influence but may however speculate that the expression of these two genes may act as a marker for CoQ activity in the tissue. In addition, the endurance phenotype of the TT/NN horses, with their higher proportion of type I fibres and concurrent increased mitochondrial content, does not align with a CoQ deficiency or the potentially decreased mitochondrial capacity that this may produce.

Commonly, CoQ is present in the mitochondrial inner membrane in stoichiometric excess over other components [55–57], therefore lower CoQ concentrations may or may not have a functional effect. It has previously been observed in rat, human and *Drosophila* that mitochondrial electron transport chain complexes appear to have ‘thresholds’, and as such they can occasionally lose a certain amount of activity without it having any overall functional impact on the capacity of mitochondria to perform oxidative phosphorylation [58–60]. For example, in nonsynaptic mitochondria isolated from rat brain, individual electron transport chain complexes I, III and IV could be inhibited by approximately 72%, 70% and 60%, respectively, prior to any significant alterations in mitochondrial respiration or ATP synthesis occurring [58]. These ‘threshold’ effects have not been extensively studied in equine skeletal muscle mitochondria; however, a recent publication which assessed the effects of aging on mitochondrial function observed a possible example of this in American Quarter horses [61]. Significantly decreased cytochrome *c* oxidase activity, indicative of decreased oxidative capacity, was observed in aged horses, without a concurrent alteration in mitochondrial respiration. This concept could play a factor in whether or not the associations noted in the present study impact on mitochondrial function.

Dietary CoQ uptake is limited in mammals [62, 63],with the majority of CoQ is endogenously synthesised. The biosynthetic process occurs in a number of locations within the cell and involves a number of common pathways. Although the full biosynthetic pathway is not yet fully understood, it is known that the terminal rate-limiting steps of CoQ biosynthesis occur in the mitochondria [64, 65] and are modulated by nuclear encoded genes specific to CoQ biosynthesis. Mutations in the genes encoding enzymes involved in the biosynthesis of CoQ have a variety of phenotypic consequences, ranging from mild to severe symptoms such as encephalomyopathy and cerebellar ataxia [66]. The severity of symptoms may be related to the level of biosynthetic impairment and hence level of CoQ deficiency, the more severe the impairment the more severe the symptoms [67]. Secondary CoQ deficiencies due to mutations in genes unrelated to CoQ biosynthesis are also associated with ataxia and mitochondrial encephalomyopathies [68–70]. Deficiencies in CoQ in skeletal muscle and the resulting myopathies have been associated with mutations in a number of the CoQ biosynthetic genes such as COQ9 [71]. However, in many cases where decreased CoQ levels in skeletal muscle were detected in association with a CoQ biosynthesis gene mutation no myopathic symptoms are observed [47, 48]. Supplementation with CoQ to patients displaying marked deficiency in the molecule have had varied results [66, 72]. In many cases oral supplementation with high doses of CoQ_10_ leads to increased CoQ levels and stops the progression of the disease.

In that regard, CoQ has been suggested as a therapeutic supplement with potential nutritional [72, 73], performance [74] and anti-fatigue benefits [75–77]. Previous studies have implicated CoQ supplementation in the alteration of fibre proportions in skeletal muscle tissue in both humans and rats [78, 79]. In humans CoQ_10_ supplementation has been used successfully as an adjunctive therapy in the treatment of congestive heart failure, muscular dystrophy and myopathies [80–83]. There is some potential for CoQ_10_ supplementation to reduce exercise-induced oxidation [75], as evidenced by a reduction in pro-oxidative biomarkers and an increase in anti-oxidant enzymes. There may also be an anti-fatigue effect of CoQ_10_ during exercise with a decrease in the rate of perceived exertion noted with CoQ_10_ supplementation [77]. A slight decrease in biomarkers of muscle damage has been noted with CoQ_10_ supplementation as well as an increase in VO_2max_ of untrained humans [84], although other studies found no influence of supplementation on oxygen uptake during exercise [85, 86]. No significant influence on anaerobic cardiovascular exercise or exercise capacity has been detected with CoQ_10_ supplementation. There are some reports of the use of CoQ_10_ in the horse that indicate no significant side-effects of the supplement [87, 88], significant reduction in pro-inflammatory gene expression [89] and a reduction in CoQ_10_ depletion following supplementation and high-intensity exercise [88]. The connection between myostatin and CoQ_10_, which results in these associations with *MSTN* genotype is, at present, unclear. Although future studies will be required to elucidate the functional effect of CoQ_10_ on Thoroughbred skeletal muscle, the findings of the present study suggest a means by which the supplementation of CoQ_10_ in horses could be individualised to those that would benefit most.

## Conclusions

*MSTN* genotype in untrained Thoroughbred horses is associated with muscle fibre proportion and as a consequence mitochondrial abundance. These phenotypes manifest in variation in endurance related performance that have a greater requirement for oxidative energy production. We have found that despite the greater oxidative requirements, TT/NN horses have significantly lower mitochondrial combined complex I+III and II+III activity, an indicator of CoQ levels, than CC/II horses. In addition, we observed a significant association between *MSTN* genotype and the expression of two CoQ biosynthetic pathway genes, *COQ4* and *ADCK3*, which may suggest decreased biosynthesis of CoQ. These data suggest that TT/NN horses may benefit from dietary supplementation of CoQ_10_, which has been shown to have a range of health benefits relating to exercise.

## Supporting information

S1 Fig Body weight (kg) and body weight/withers height (kg/cm) of subset of Thoroughbred horses biopsied near the time of untrained biopsy compared on the basis of three *MSTN* g.66493737C>T SNP/SINE insertion 227 bp genotypes (CC/II, CT/IN, TT/NN). Body weight (to the nearest kg) and withers height (to the nearest cm) was measured for each Thoroughbred horse used in the study, the figures shown above include only those where parameters (body weight and withers height) were measured within 60 days of skeletal muscle biopsy. Body weight in kilograms (A) and body weight/withers height in kilograms per centimetre (B) were compared between the three *MSTN* g.66493737C>T SNP/SINE insertion 227bp genotypes (CC/II: n = 24, CT/IN: n = 17 and TT/NN; n = 6). Results presented with mean ± SEM, p-values where shown indicate significance as measured by a one-way ANOVA with a Bonferroni multiple comparison post-test, * = p ≤ 0.05, ** = p ≤ 0.01, *** = p ≤ 0.001.
(TIF)

S2 Fig Submaximal training pre-biopsy of untrained Thoroughbred horses of three *MSTN* g.66493737C>T SNP/SINE insertion 227 bp genotypes (CC/II, CT/IN, TT/NN). Information in relation to the amount of submaximal exercise the horses within the study did prior to the *gluteus medius* biopsy was gathered. (A) Shows the number of days between breaking (teaching the horse to be ridden) to the date of biopsy and (B) shows the number of days between the date of first canter (slow exercise) to the date of biopsy. All horses in the study were included (CC/II: n = 37, CT/IN: n = 34 and TT/NN; n = 11). Results presented with mean ± SEM, p-values where shown indicate significance as measured by a one-way ANOVA with a Bonferroni multiple comparison post-test.
(TIF)

S3 Fig Expression of CoQ biosynthesis genes in *MSTN* genotype (CC/II, CT/IN, TT/NN) horses. qPCR was used to measure *PDSS1* (A), *PDSS2* (B), *COQ2* (C), *COQ3* (D), *COQ5* (E), *COQ6* (F), *COQ7* (G), *COQ9* (H) and *ADCK4* (I) gene expression levels. RNA was isolated from *gluteus medius* skeletal muscle from untrained Thoroughbred horses (21±3 months), reverse transcribed into cDNA and amplified using specific primers in real-time PCR; CC/II: n = 36, CT/IN: n = 34 and TT/NN: n = 11, performed in at least duplicate. Gene expression was normalised to the expression of *HPRT* using the ΔΔCt method. Results presented with mean ± SEM, p-values where shown indicate significance as measured by a one-way ANOVA with a Bonferroni multiple comparison post-test.
(TIF)

S4 Fig CoQ biosynthesis enzymes protein expression in *MSTN* genotype (CC/II, CT/IN, TT/NN) horses. COQ4, ADCK3 and COQ3 protein levels were measured by western blot analysis in untrained Thoroughbred skeletal muscle protein lysates of three *MSTN* genotypes. A representative experiment out of six performed is shown for each protein. Densitometry was performed and corresponding results are presented with mean ± SEM, p-values where shown indicate significance as measured by a one-way ANOVA with a Bonferroni multiple comparison post-test.
(TIF)

S1 Table Primer sequences for real-time qPCR. (TIF)

S2 Table Citrate synthase and electron transport chain complex activity values for Thoroughbred horse skeletal muscle tissue. Table of mean ± standard error of the mean (n) values for all spectrophotometric assays. Spectrophotometric results are based on experiments performed in at least duplicate for each sample.
(TIF)

## References

[1] McPherron AC, Lawler AM, Lee SJ. Regulation of skeletal muscle mass in mice by a new TGF-beta superfamily member. Nature. 1997;387(6628):83–90. Epub 1997/05/01. doi: 10.1038/387083a0 . 9139826 10.1038/387083a0

[2] Dominique J-E, Gérard C. Myostatin regulation of muscle development: molecular basis, natural mutations, physiopathological aspects. Experimental cell research. 2006;312(13):2401–14. doi: 10.1016/j.yexcr.2006.04.012 16793037 10.1016/j.yexcr.2006.04.012

[3] Zhu X, Hadhazy M, Wehling M, Tidball JG, McNally EM. Dominant negative myostatin produces hypertrophy without hyperplasia in muscle. FEBS letters. 2000;474(1):71–5. 10828454 10.1016/s0014-5793(00)01570-2

[4] Yang J, Ratovitski T, Brady JP, Solomon MB, Wells KD, Wall RJ. Expression of myostatin pro domain results in muscular transgenic mice. Molecular reproduction and development. 2001;60(3):351–61. doi: 10.1002/mrd.1097 11599046 10.1002/mrd.1097

[5] Girgenrath S, Song K, Whittemore LA. Loss of myostatin expression alters fiber-type distribution and expression of myosin heavy chain isoforms in slow- and fast-type skeletal muscle. Muscle & nerve. 2005;31(1):34–40. Epub 2004/10/07. doi: 10.1002/mus.20175 . 15468312 10.1002/mus.20175

[6] Amthor H, Macharia R, Navarrete R, Schuelke M, Brown SC, Otto A, et al Lack of myostatin results in excessive muscle growth but impaired force generation. Proceedings of the National Academy of Sciences. 2007;104(6):1835–40. Epub 2007/02/03. doi: 10.1073/pnas.0604893104 ; PubMed Central PMCID: PMC1794294. 17267614 10.1073/pnas.0604893104 PMC1794294

[7] Ploquin C, Chabi B, Fouret G, Vernus B, Feillet-Coudray C, Coudray C, et al Lack of myostatin alters intermyofibrillar mitochondria activity, unbalances redox status, and impairs tolerance to chronic repetitive contractions in muscle. American journal of physiology-Endocrinology and metabolism. 2012;302(8):E1000–8. Epub 2012/02/10. doi: 10.1152/ajpendo.00652.2011 . 22318951 10.1152/ajpendo.00652.2011

[8] Mathieu O, Krauer R, Hoppeler H, Gehr P, Lindstedt SL, Alexander RM, et al Design of the mammalian respiratory system. VII. Scaling mitochondrial volume in skeletal muscle to body mass. Respiration physiology. 1981;44(1):113–28. Epub 1981/04/01. . 7232882 10.1016/0034-5687(81)90079-7

[9] Cunningham E, Dooley J, Splan R, Bradley D. Microsatellite diversity, pedigree relatedness and the contributions of founder lineages to thoroughbred horses. Animal genetics. 2001;32(6):360–4. 11736806 10.1046/j.1365-2052.2001.00785.x

[10] Hill EW, Bradley DG, Al-Barody M, Ertugrul O, Splan RK, Zakharov I, et al History and integrity of thoroughbred dam lines revealed in equine mtDNA variation. Animal genetics. 2002;33(4):287–94. Epub 2002/07/26. . 12139508 10.1046/j.1365-2052.2002.00870.x

[11] Bower MA, Campana MG, Whitten M, Edwards CJ, Jones H, Barrett E, et al The cosmopolitan maternal heritage of the Thoroughbred racehorse breed shows a significant contribution from British and Irish native mares. Biology letters. 2011;7(2):316–20. Epub 2010/10/12. doi: 10.1098/rsbl.2010.0800 ; PubMed Central PMCID: PMC3061175. 20926431 10.1098/rsbl.2010.0800 PMC3061175

[12] Jones JH, Longworth KE, Lindholm A, Conley KE, Karas RH, Kayar SR, et al Oxygen transport during exercise in large mammals. I. Adaptive variation in oxygen demand. Journal of applied physiology. 1989;67(2):862–70. Epub 1989/08/01. . 2793686 10.1152/jappl.1989.67.2.862

[13] Jones JH, Lindstedt S. Limits to maximal performance. Annual review of physiology. 1993;55(1):547–69. 10.1146/annurev.ph.55.030193.002555 8466184

[14] Young LE, Marlin DJ, Deaton C, Brown-Feltner H, Roberts CA, Wood JL. Heart size estimated by echocardiography correlates with maximal oxygen uptake. Equine veterinary journal, Supplement. 2002;(34):467–71. Epub 2002/10/31. doi: 10.1111/j.2042-3306.2002.tb05467.x . 12405735 10.1111/j.2042-3306.2002.tb05467.x

[15] Kayar S, Hoppeler H, Lindstedt S, Claassen H, Jones J, Essen-Gustavsson B, et al Total muscle mitochondrial volume in relation to aerobic capacity of horses and steers. Pflügers Archiv. 1989;413(4):343–7. 2928085 10.1007/BF00584481

[16] Binns M, Boehler D, Lambert D. Identification of the myostatin locus (MSTN) as having a major effect on optimum racing distance in the Thoroughbred horse in the USA. Animal genetics. 2010;41(s2):154–8. 21070290 10.1111/j.1365-2052.2010.02126.x

[17] Hill EW, Gu J, Eivers SS, Fonseca RG, McGivney BA, Govindarajan P, et al A sequence polymorphism in MSTN predicts sprinting ability and racing stamina in thoroughbred horses. PloS one. 2010;5(1):e8645 Epub 2010/01/26. doi: 10.1371/journal.pone.0008645 ; PubMed Central PMCID: PMC2808334. 20098749 10.1371/journal.pone.0008645 PMC2808334

[18] Hill EW, McGivney BA, Gu J, Whiston R, Machugh DE. A genome-wide SNP-association study confirms a sequence variant (g.66493737C>T) in the equine myostatin (MSTN) gene as the most powerful predictor of optimum racing distance for Thoroughbred racehorses. BMC genomics. 2010;11:552 Epub 2010/10/12. doi: 10.1186/1471-2164-11-552 ; PubMed Central PMCID: PMC3091701. 20932346 10.1186/1471-2164-11-552 PMC3091701

[19] Tozaki T, Miyake T, Kakoi H, Gawahara H, Sugita S, Hasegawa T, et al A genome‐wide association study for racing performances in Thoroughbreds clarifies a candidate region near the MSTN gene. Animal genetics. 2010;41(s2):28–35. 21070273 10.1111/j.1365-2052.2010.02095.x

[20] van den Hoven R, Gür E, Schlamanig M, Hofer M, Onmaz AC, Steinborn R. Putative regulation mechanism for the MSTN gene by a CpG island generated by the SINE marker Ins227bp. BMC veterinary research. 2015;11(1):1. 26100061 10.1186/s12917-015-0428-3 PMC4476204

[21] Tozaki T, Sato F, Hill EW, Miyake T, Endo Y, Kakoi H, et al Sequence variants at the myostatin gene locus influence the body composition of Thoroughbred horses. The Journal of veterinary medical science / the Japanese Society of Veterinary Science. 2011;73(12):1617–24. Epub 2011/08/13. . 21836385 10.1292/jvms.11-0295

[22] Petersen JL, Mickelson JR, Rendahl AK, Valberg SJ, Andersson LS, Axelsson J, et al Genome-wide analysis reveals selection for important traits in domestic horse breeds. PLoS genetics. 2013;9(1):e1003211 doi: 10.1371/journal.pgen.1003211 23349635 10.1371/journal.pgen.1003211 PMC3547851

[23] Petersen JL, Valberg SJ, Mickelson JR, McCue ME. Haplotype diversity in the equine myostatin gene with focus on variants associated with race distance propensity and muscle fiber type proportions. Animal genetics. 2014 Epub 2014/08/28. doi: 10.1111/age.12205 . 25160752 10.1111/age.12205 PMC4211974

[24] Rivero JL, Serrano AL, Barrey E, Valette JP, Jouglin M. Analysis of myosin heavy chains at the protein level in horse skeletal muscle. Journal of Muscle Research & Cell Motility. 1999;20(2):211–21. 10412092 10.1023/a:1005461214800

[25] Ledwith A, McGowan CM. Muscle biopsy: a routine diagnostic procedure. Equine Veterinary Education. 2004;16(2):62–7.

[26] Hill EW, Fonseca RG, McGivney BA, Gu J, MacHugh DE, Katz LM. MSTN genotype (g.66493737C/T) association with speed indices in Thoroughbred racehorses. Journal of applied physiology. 2012;112(1):86–90. Epub 2011/10/22. doi: 10.1152/japplphysiol.00793.2011 . 22016373 10.1152/japplphysiol.00793.2011

[27] Smith PK, Krohn RI, Hermanson GT, Mallia AK, Gartner FH, Provenzano MD, et al Measurement of protein using bicinchoninic acid. Analytical biochemistry. 1985;150(1):76–85. Epub 1985/10/01. . 3843705 10.1016/0003-2697(85)90442-7

[28] Srere PA. [1] Citrate synthase: [EC 4.1.3.7. Citrate oxaloacetate-lyase (CoA-acetylating)] In: John ML, editor. Methods in Enzymology. Volume 13: Academic Press; 1969 p. 3–11.

[29] Ragan C, Wilson M, Darley-Usmar V, Lowe P. Subfractionation of mitochondria and isolation of the proteins of oxidative phosphorylation. Mitochondria: a practical approach. 1987:79–112.

[30] Hatefi Y. [6] Resolution of complex II and isolation of succinate dehydrogenase (EC 1.3. 99.1). Methods in enzymology. 1978;53:27–35. 713837 10.1016/s0076-6879(78)53009-7

[31] Wharton DC, Tzagoloff A. [45] Cytochrome oxidase from beef heart mitochondria. Methods in enzymology. 1967;10:245–50.

[32] Powers WJ, Haas RH, Le T, Videen TO, Hershey T, McGee-Minnich L, et al Normal platelet mitochondrial complex I activity in Huntington’s disease. Neurobiology of disease. 2007;27(1):99–101. doi: 10.1016/j.nbd.2007.04.008 17543533 10.1016/j.nbd.2007.04.008 PMC2140002

[33] King TE. [40] Preparations of succinate—cytochrome c reductase and the cytochrome bc 1 particle, and reconstitution of succinate-cytochrome c reductase. Methods in enzymology. 1967;10:216–25.

[34] Untergasser A, Nijveen H, Rao X, Bisseling T, Geurts R, Leunissen JA. Primer3Plus, an enhanced web interface to Primer3. Nucleic acids research. 2007;35(suppl 2):W71–W4. 17485472 10.1093/nar/gkm306 PMC1933133

[35] Hemmings KM, Parr T, Daniel ZC, Picard B, Buttery PJ, Brameld JM. Examination of myosin heavy chain isoform expression in ovine skeletal muscles. Journal of animal science. 2009;87(12):3915–22. Epub 2009/08/18. doi: 10.2527/jas.2009-2067 . 19684280 10.2527/jas.2009-2067

[36] Altschul SF, Gish W, Miller W, Myers EW, Lipman DJ. Basic local alignment search tool. Journal of molecular biology. 1990;215(3):403–10. doi: 10.1016/S0022-2836(05)80360-2 2231712 10.1016/S0022-2836(05)80360-2

[37] Cappelli K, Felicetti M, Capomaccio S, Spinsanti G, Silvestrelli M, Supplizi AV. Exercise induced stress in horses: selection of the most stable reference genes for quantitative RT-PCR normalization. BMC molecular biology. 2008;9(1):49. 18489742 10.1186/1471-2199-9-49 PMC2412902

[38] Laemmli UK. Cleavage of structural proteins during the assembly of the head of bacteriophage T4. Nature. 1970;227(5259):680–5. Epub 1970/08/15. . 5432063 10.1038/227680a0

[39] Schneider CA, Rasband WS, Eliceiri KW. NIH Image to ImageJ: 25 years of image analysis. Nature methods. 2012;9(7):671–5. 22930834 10.1038/nmeth.2089 PMC5554542

[40] Kirby DM, Thorburn DR, Turnbull DM, Taylor RW. Biochemical assays of respiratory chain complex activity. Methods in cell biology. 2007;80:93–119. doi: 10.1016/S0091-679X(06)80004-X 17445690 10.1016/S0091-679X(06)80004-X

[41] Jonckheere AI, Smeitink JA, Rodenburg RJ. Mitochondrial ATP synthase: architecture, function and pathology. Journal of inherited metabolic disease. 2012;35(2):211–25. doi: 10.1007/s10545-011-9382-9 21874297 10.1007/s10545-011-9382-9 PMC3278611

[42] Ogata T. A histochemical study of the red and white muscle fibers. Part 1. Activity of the succinoxydase system in muscle fibers. Acta Medica. 1958;Okayama(12):216–27.

[43] Ingjer F. Effects of endurance training on muscle fibre ATP-ase activity, capillary supply and mitochondrial content in man. The Journal of Physiology. 1979;294(1):419–32. 159945 10.1113/jphysiol.1979.sp012938 PMC1280565

[44] Greene HM, Wickler SJ, Tucker RL, London C. Fiber type composition of the middle gluteal muscle of mules. Journal of Equine Veterinary Science. 1995;15(9):388–91. http://dx.doi.org/10.1016/S0737-0806(07)80482-5.

[45] Zierath JR, Hawley JA. Skeletal muscle fiber type: influence on contractile and metabolic properties. PLoS Biol. 2004;2(10):e348 doi: 10.1371/journal.pbio.0020348 15486583 10.1371/journal.pbio.0020348 PMC521732

[46] Serrano A, Quiroz-Rothe E, Rivero J-L. Early and long-term changes of equine skeletal muscle in response to endurance training and detraining. Pflügers Archiv. 2000;441(2–3):263–74. 11211112 10.1007/s004240000408

[47] Lagier-Tourenne C, Tazir M, Lopez LC, Quinzii CM, Assoum M, Drouot N, et al ADCK3, an ancestral kinase, is mutated in a form of recessive ataxia associated with coenzyme Q10 deficiency. American journal of human genetics. 2008;82(3):661–72. Epub 2008/03/06. doi: 10.1016/j.ajhg.2007.12.024 ; PubMed Central PMCID: PMCPMC2427193. 18319074 10.1016/j.ajhg.2007.12.024 PMC2427193

[48] Mollet J, Delahodde A, Serre V, Chretien D, Schlemmer D, Lombes A, et al CABC1 gene mutations cause ubiquinone deficiency with cerebellar ataxia and seizures. The American Journal of Human Genetics. 2008;82(3):623–30. doi: 10.1016/j.ajhg.2007.12.022 18319072 10.1016/j.ajhg.2007.12.022 PMC2427298

[49] Salviati L, Trevisson E, Hernandez MAR, Casarin A, Pertegato V, Doimo M, et al Haploinsufficiency of COQ4 causes coenzyme Q10 deficiency. Journal of medical genetics. 2012;49(3):187–91. doi: 10.1136/jmedgenet-2011-100394 22368301 10.1136/jmedgenet-2011-100394 PMC3983946

[50] Vogel C, Marcotte EM. Insights into the regulation of protein abundance from proteomic and transcriptomic analyses. Nature Reviews Genetics. 2012;13(4):227–32. doi: 10.1038/nrg3185 22411467 10.1038/nrg3185 PMC3654667

[51] de Sousa Abreu R, Penalva LO, Marcotte EM, Vogel C. Global signatures of protein and mRNA expression levels. Molecular BioSystems. 2009;5(12):1512–26. doi: 10.1039/b908315d 20023718 10.1039/b908315d PMC4089977

[52] Maier T, Güell M, Serrano L. Correlation of mRNA and protein in complex biological samples. FEBS letters. 2009;583(24):3966–73. doi: 10.1016/j.febslet.2009.10.036 19850042 10.1016/j.febslet.2009.10.036

[53] Schwanhäusser B, Busse D, Li N, Dittmar G, Schuchhardt J, Wolf J, et al Global quantification of mammalian gene expression control. Nature. 2011;473(7347):337–42. doi: 10.1038/nature10098 21593866 10.1038/nature10098

[54] Vogel C, de Sousa Abreu R, Ko D, Le SY, Shapiro BA, Burns SC, et al Sequence signatures and mRNA concentration can explain two‐thirds of protein abundance variation in a human cell line. Molecular systems biology. 2010;6(1):400. 20739923 10.1038/msb.2010.59 PMC2947365

[55] Vinogradov A, King TE. [15] The Keilin-Hartree heart muscle preparation. Methods in enzymology. 1979;55:118–27. 156830 10.1016/0076-6879(79)55017-4

[56] Capaldi RA. Arrangement of proteins in the mitochondrial inner membrane. Biochimica et Biophysica Acta (BBA)-Reviews on Biomembranes. 1982;694(3):291–306. 6295486 10.1016/0304-4157(82)90009-0

[57] Slater E. The mechanism of the conservation of energy of biological oxidations. The FEBS Journal. 1987;166(3):489–504. 10.1111/j.1432-1033.1987.tb13542.x 3038543

[58] Davey GP, Clark JB. Threshold effects and control of oxidative phosphorylation in nonsynaptic rat brain mitochondria. Journal of neurochemistry. 1996;66(4):1617–24. 8627318 10.1046/j.1471-4159.1996.66041617.x

[59] Farge G, Touraille S, Debise R, Alziari S. The respiratory chain complex thresholds in mitochondria of a Drosophila subobscura mutant strain. Biochimie. 2002;84(12):1189–97. 12628295 10.1016/s0300-9084(02)00038-x

[60] Rossignol R, Faustin B, Rocher C, Malgat M, Mazat J-P, Letellier T. Mitochondrial threshold effects. Biochemical Journal. 2003;370(3):751–62. 12467494 10.1042/BJ20021594 PMC1223225

[61] Li C, White SH, Warren LK, Wohlgemuth SE. Effects of aging on mitochondrial function in skeletal muscle of American Quarter Horses. Journal of Applied Physiology. 2016;121(1):299–311. doi: 10.1152/japplphysiol.01077.2015 27283918 10.1152/japplphysiol.01077.2015 PMC5040552

[62] Bergamini C, Moruzzi N, Sblendido A, Lenaz G, Fato R. A water soluble CoQ 10 formulation improves intracellular distribution and promotes mitochondrial respiration in cultured cells. PloS one. 2012;7(3):e33712 doi: 10.1371/journal.pone.0033712 22432044 10.1371/journal.pone.0033712 PMC3303850

[63] Bhagavan HN, Chopra RK. Plasma coenzyme Q10 response to oral ingestion of coenzyme Q10 formulations. Mitochondrion. 2007;7:S78–S88. doi: 10.1016/j.mito.2007.03.003 17482886 10.1016/j.mito.2007.03.003

[64] Gin P, Clarke CF. Genetic evidence for a multi-subunit complex in coenzyme Q biosynthesis in yeast and the role of the Coq1 hexaprenyl diphosphate synthase. Journal of Biological Chemistry. 2005;280(4):2676–81. doi: 10.1074/jbc.M411527200 15548532 10.1074/jbc.M411527200

[65] Johnson A, Gin P, Marbois BN, Hsieh EJ, Wu M, Barros MH, et al COQ9, a new gene required for the biosynthesis of coenzyme Q in Saccharomyces cerevisiae. Journal of Biological Chemistry. 2005;280(36):31397–404. doi: 10.1074/jbc.M503277200 16027161 10.1074/jbc.M503277200

[66] Desbats MA, Lunardi G, Doimo M, Trevisson E, Salviati L. Genetic bases and clinical manifestations of coenzyme Q10 (CoQ10) deficiency. Journal of inherited metabolic disease. 2015;38(1):145–56. doi: 10.1007/s10545-014-9749-9 25091424 10.1007/s10545-014-9749-9

[67] Trevisson E, DiMauro S, Navas P, Salviati L. Coenzyme Q deficiency in muscle. Current opinion in neurology. 2011;24(5):449–56. doi: 10.1097/WCO.0b013e32834ab528 21844807 10.1097/WCO.0b013e32834ab528

[68] Quinzii C, Kattah A, Naini A, Akman H, Mootha V, DiMauro S, et al Coenzyme Q deficiency and cerebellar ataxia associated with an aprataxin mutation. Neurology. 2005;64(3):539–41. doi: 10.1212/01.WNL.0000150588.75281.58 15699391 10.1212/01.WNL.0000150588.75281.58

[69] Miles MV, Miles L, Tang PH, Horn PS, Steele PE, DeGrauw AJ, et al Systematic evaluation of muscle coenzyme Q10 content in children with mitochondrial respiratory chain enzyme deficiencies. Mitochondrion. 2008;8(2):170–80. doi: 10.1016/j.mito.2008.01.003 18313367 10.1016/j.mito.2008.01.003

[70] Sacconi S, Trevisson E, Salviati L, Aymé S, Rigal O, Redondo AG, et al Coenzyme Q 10 is frequently reduced in muscle of patients with mitochondrial myopathy. Neuromuscular Disorders. 2010;20(1):44–8. doi: 10.1016/j.nmd.2009.10.014 19945282 10.1016/j.nmd.2009.10.014

[71] Duncan AJ, Bitner-Glindzicz M, Meunier B, Costello H, Hargreaves IP, López LC, et al A nonsense mutation in COQ9 causes autosomal-recessive neonatal-onset primary coenzyme Q 10 deficiency: a potentially treatable form of mitochondrial disease. The American Journal of Human Genetics. 2009;84(5):558–66. doi: 10.1016/j.ajhg.2009.03.018 19375058 10.1016/j.ajhg.2009.03.018 PMC2681001

[72] Borekova M, Hojerova J, Koprda V, Bauerova K. Nourishing and health benefits of coenzyme Q10-a review. Czech journal of food sciences. 2008;26(4):229–41.

[73] Littarru GP, Tiano L. Clinical aspects of coenzyme Q 10: an update. Nutrition. 2010;26(3):250–4. doi: 10.1016/j.nut.2009.08.008 19932599 10.1016/j.nut.2009.08.008

[74] Cooke M, Iosia M, Buford T, Shelmadine B, Hudson G, Kerksick C, et al Effects of acute and 14-day coenzyme Q10 supplementation on exercise performance in both trained and untrained individuals. Journal of the International Society of Sports Nutrition. 2008;5(1):1. 18318910 10.1186/1550-2783-5-8 PMC2315638

[75] Kon M, Kimura F, Akimoto T, Tanabe K, Murase Y, Ikemune S, et al Effect of Coenzyme Q10 supplementation on exercise-induced muscular injury of rats. Exercise immunology review. 2007;13:76–88. Epub 2008/01/18. . 18198662

[76] Kon M, Tanabe K, Akimoto T, Kimura F, Tanimura Y, Shimizu K, et al Reducing exercise-induced muscular injury in kendo athletes with supplementation of coenzyme Q 10. British journal of nutrition. 2008;100(04):903–9. 18284711 10.1017/S0007114508926544

[77] Mizuno K, Tanaka M, Nozaki S, Mizuma H, Ataka S, Tahara T, et al Antifatigue effects of coenzyme Q10 during physical fatigue. Nutrition. 2008;24(4):293–9. doi: 10.1016/j.nut.2007.12.007 18272335 10.1016/j.nut.2007.12.007

[78] Linnane A, Degli Esposti M, Generowicz M, Luff A, Nagley P. The universality of bioenergetic disease and amelioration with redox therapy. Biochimica et Biophysica Acta (BBA)-Molecular Basis of Disease. 1995;1271(1):191–4. 7599207 10.1016/0925-4439(95)00027-2

[79] Linnane AW, Kopsidas G, Zhang C, Yarovaya N, Kovalenko S, Papakostopoulos P, et al Cellular redox activity of coenzyme Q 10: effect of CoQ 10 supplementation on human skeletal muscle. Free radical research. 2002;36(4):445–53. 12069109 10.1080/10715760290021306

[80] Morisco C, Trimarco B, Condorelli M. Effect of coenzyme Q10 therapy in patients with congestive heart failure: a long-term multicenter randomized study. The clinical investigator. 1993;71(8):S134–S6. 8241697 10.1007/BF00226854

[81] Folkers K, Simonsen R. Two successful double-blind trials with coenzyme Q10 (vitamin Q10) on muscular dystrophies and neurogenic atrophies. Biochimica et Biophysica Acta (BBA)-Molecular Basis of Disease. 1995;1271(1):281–6. 7599221 10.1016/0925-4439(95)00040-b

[82] Caso G, Kelly P, McNurlan MA, Lawson WE. Effect of coenzyme q10 on myopathic symptoms in patients treated with statins. The American journal of cardiology. 2007;99(10):1409–12. doi: 10.1016/j.amjcard.2006.12.063 17493470 10.1016/j.amjcard.2006.12.063

[83] Spurney CF, Rocha CT, Henricson E, Florence J, Mayhew J, Gorni K, et al CINRG pilot trial of coenzyme Q10 in steroid‐treated duchenne muscular dystrophy. Muscle & nerve. 2011;44(2):174–8. 21698649 10.1002/mus.22047 PMC3136634

[84] Bonetti A, Solito F, Carmosino G, Bargossi A, Fiorella P. Effect of ubidecarenone oral treatment on aerobic power in middle-aged trained subjects. Journal of Sports Medicine and Physical Fitness. 2000;40(1):51 10822909

[85] Braun B, Clarkson PM, Freedson PS, Kohl RL. Effects of coenzyme Q10 supplementation on exercise performance, VO 2 max, and lipid peroxidation in trained cyclists. International Journal of Sport Nutrition. 1991;1(4):353–65. 1844568 10.1123/ijsn.1.4.353

[86] Snider IP, Bazzarre TL, Murdoch SD, Goldfarb A. Effects of coenzyme athletic performance system as an ergogenic aid on endurance performance to exhaustion. International journal of sport nutrition. 1992;2(3):272–86. 1338584 10.1123/ijsn.2.3.272

[87] Sinatra ST, Chopra RK, Jankowitz S, Horohov DW, Bhagavan HN. Coenzyme Q10 in equine serum: response to supplementation. Journal of Equine Veterinary Science. 2013;33(2):71–3.

[88] Sinatra ST, Jankowitz SN, Chopra RK, Bhagavan HN. Plasma coenzyme Q10 and tocopherols in thoroughbred race horses: Effect of coenzyme Q10 supplementation and exercise. Journal of Equine Veterinary Science. 2013;34(2):265–9.

[89] Horohov DW, Sinatra ST, Chopra RK, Jankowitz S, Betancourt A, Bloomer RJ. The effect of exercise and nutritional supplementation on proinflammatory cytokine expression in young racehorses during training. Journal of Equine Veterinary Science. 2012;32(12):805–15.

[90] Rooney MF, Hill EW, Katz LM, Porter RK. MSTN genotype variation affects skeletal muscle mitochondrial abundance and fibre composition in untrained Thoroughbred horses. Biochimica et Biophysica Acta (BBA)—Bioenergetics. 2016;1857, Supplement:e31 http://dx.doi.org/10.1016/j.bbabio.2016.04.064.

