# Supplementary figures and images for: Skeletal muscle mitochondrial bioenergetics and associations with myostatin genotypes in the Thoroughbred horse
Source: PLoS One. 2017 Nov 30;12(11):e0186247. doi: 10.1371/journal.pone.0186247 (PMC5708611; doi:10.1371/journal.pone.0186247)

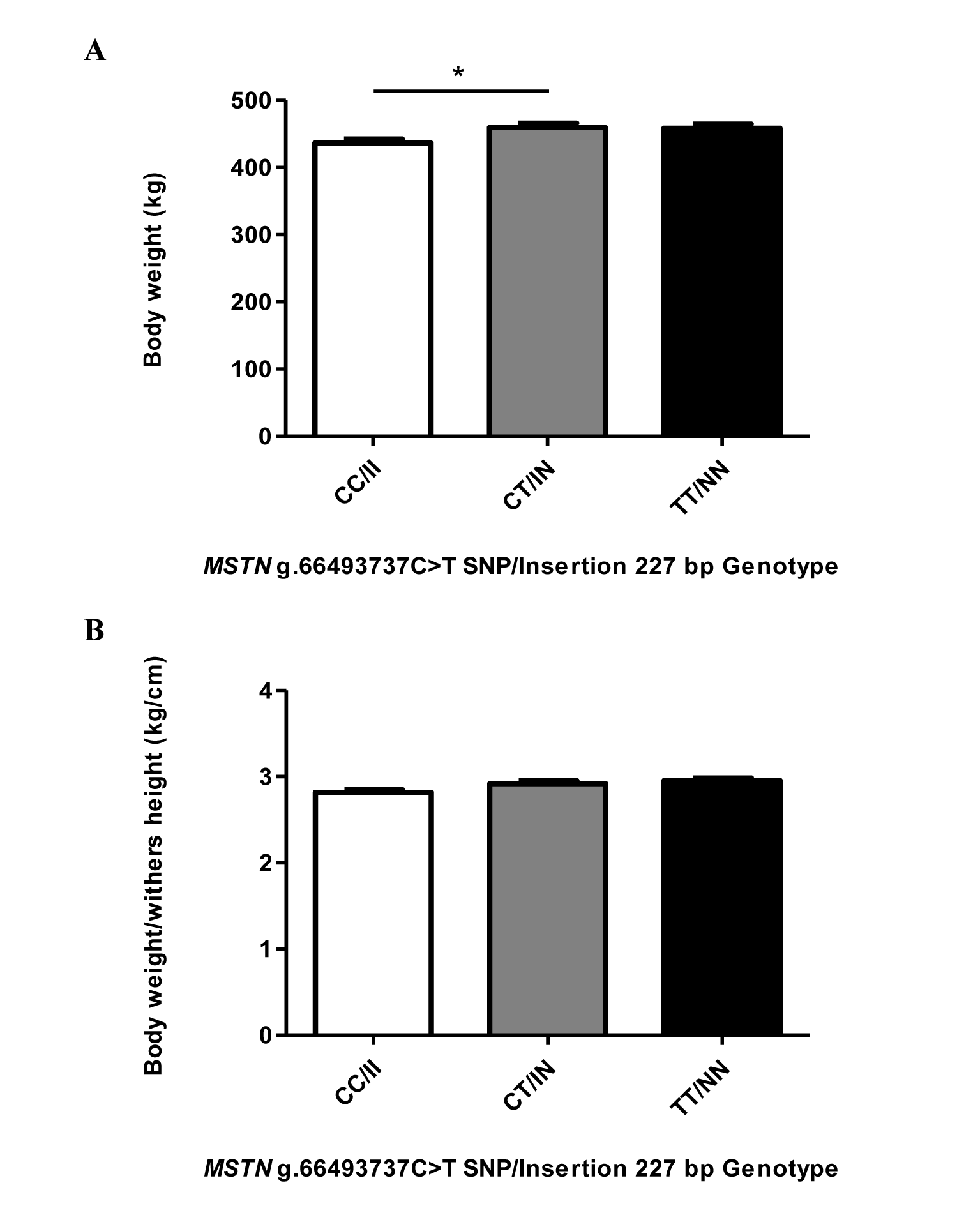

Supplement: S1 Fig — Body weight (to the nearest kg) and withers height (to the nearest cm) was measured for each Thoroughbred horse used in the study, the figures shown above include only those where parameters (body weight and withers height) were measured within 60 days of skeletal muscle biopsy. Body weight in kilograms (A) and body weight/withers height in kilograms per centimetre (B) were compared between the three MSTN g.66493737C>T SNP/SINE insertion 227bp genotypes (CC/II: n = 24, CT/IN: n = 17 and TT/NN; n = 6). Results presented with mean ± SEM, p-values where shown indicate significance as measured by a one-way ANOVA with a Bonferroni multiple comparison post-test, * = p ≤ 0.05, ** = p ≤ 0.01, *** = p ≤ 0.001. (TIF) [file pone.0186247.s001.tif]

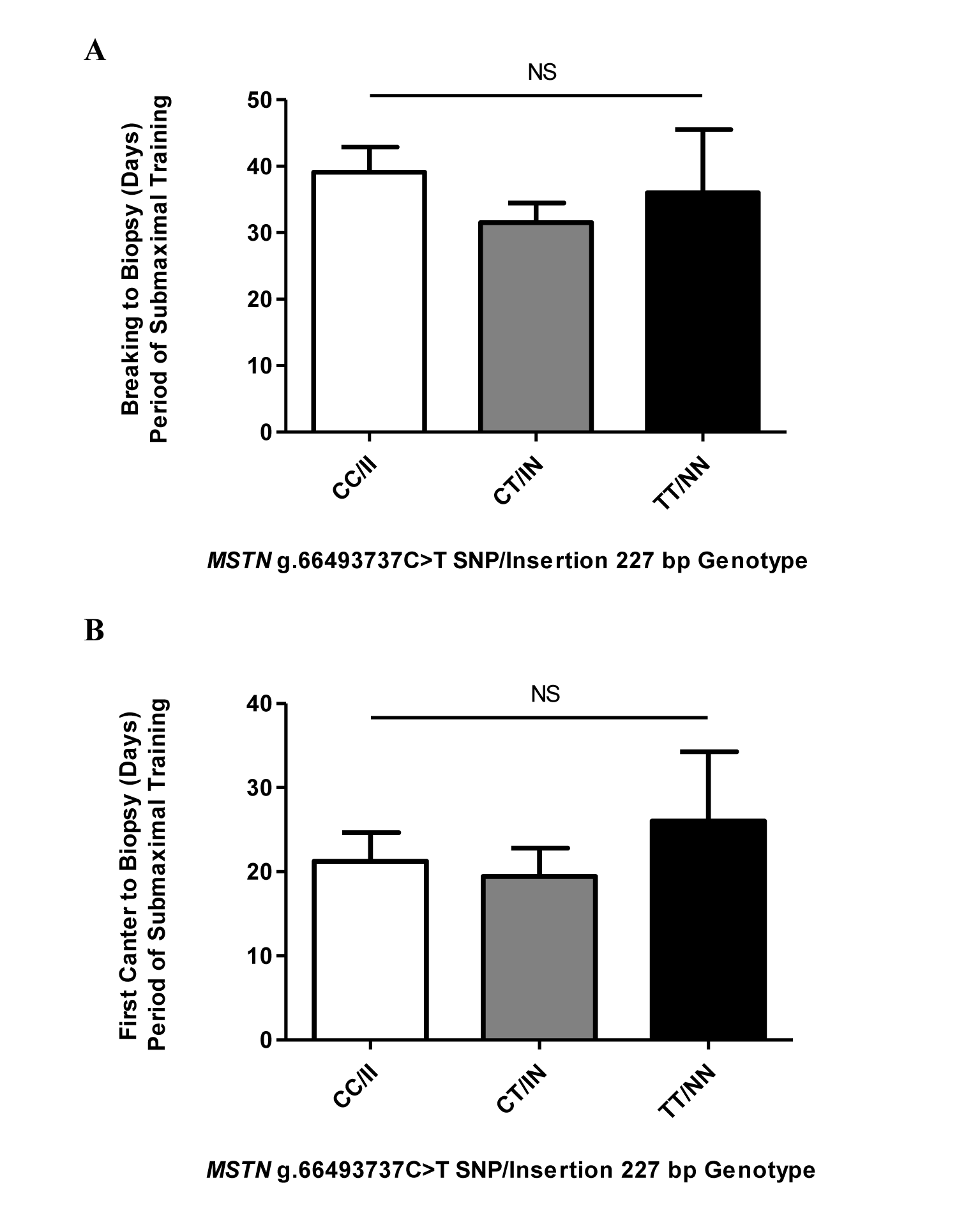

Supplement: S2 Fig — Information in relation to the amount of submaximal exercise the horses within the study did prior to the gluteus medius biopsy was gathered. (A) Shows the number of days between breaking (teaching the horse to be ridden) to the date of biopsy and (B) shows the number of days between the date of first canter (slow exercise) to the date of biopsy. All horses in the study were included (CC/II: n = 37, CT/IN: n = 34 and TT/NN; n = 11). Results presented with mean ± SEM, p-values where shown indicate significance as measured by a one-way ANOVA with a Bonferroni multiple comparison post-test. (TIF) [file pone.0186247.s002.tif]

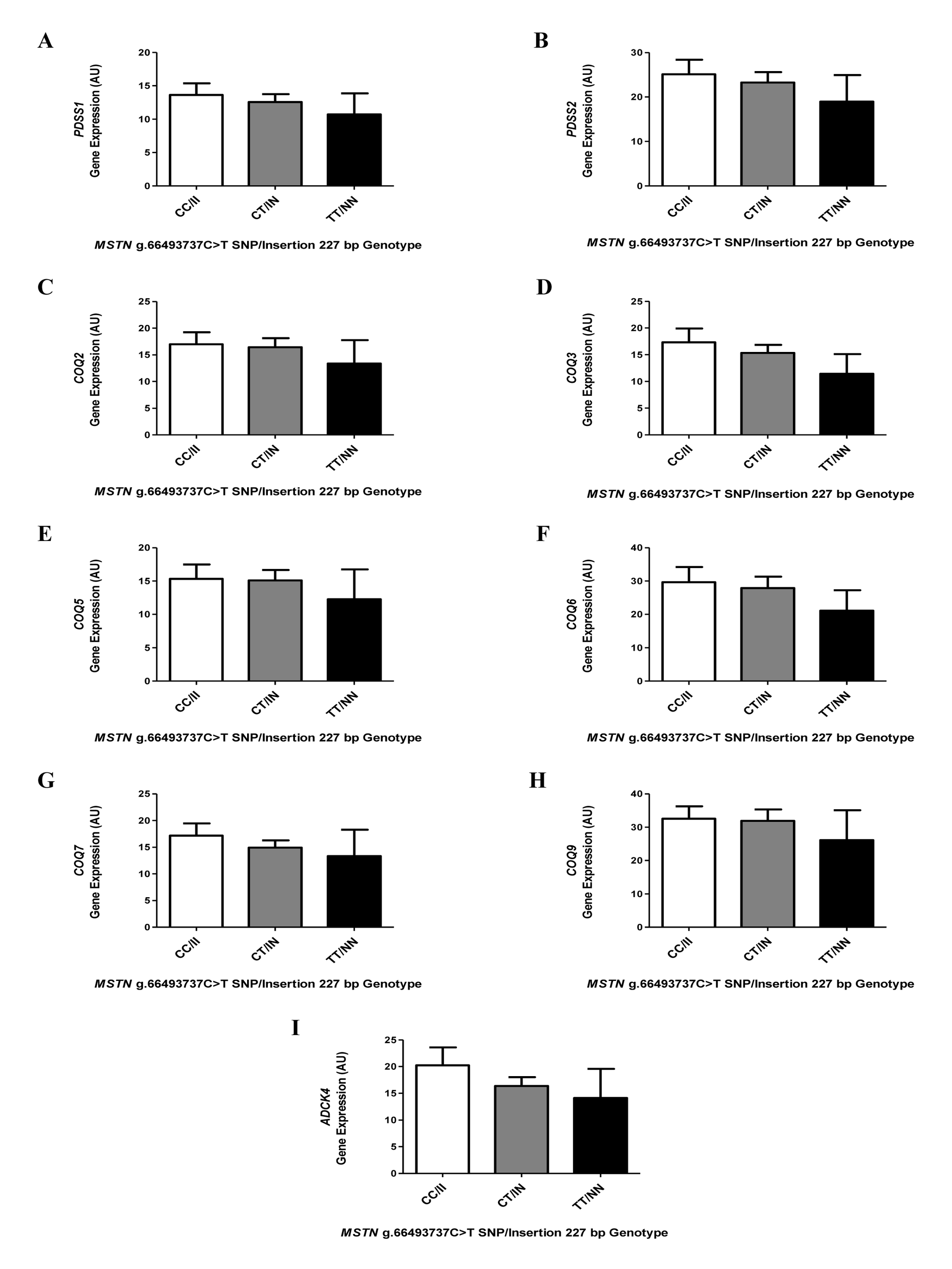

Supplement: S3 Fig — qPCR was used to measure PDSS1 (A), PDSS2 (B), COQ2 (C), COQ3 (D), COQ5 (E), COQ6 (F), COQ7 (G), COQ9 (H) and ADCK4 (I) gene expression levels. RNA was isolated from gluteus medius skeletal muscle from untrained Thoroughbred horses (21±3 months), reverse transcribed into cDNA and amplified using specific primers in real-time PCR; CC/II: n = 36, CT/IN: n = 34 and TT/NN: n = 11, performed in at least duplicate. Gene expression was normalised to the expression of HPRT using the ΔΔCt method. Results presented with mean ± SEM, p-values where shown indicate significance as measured by a one-way ANOVA with a Bonferroni multiple comparison post-test. (TIF) [file pone.0186247.s003.tif]

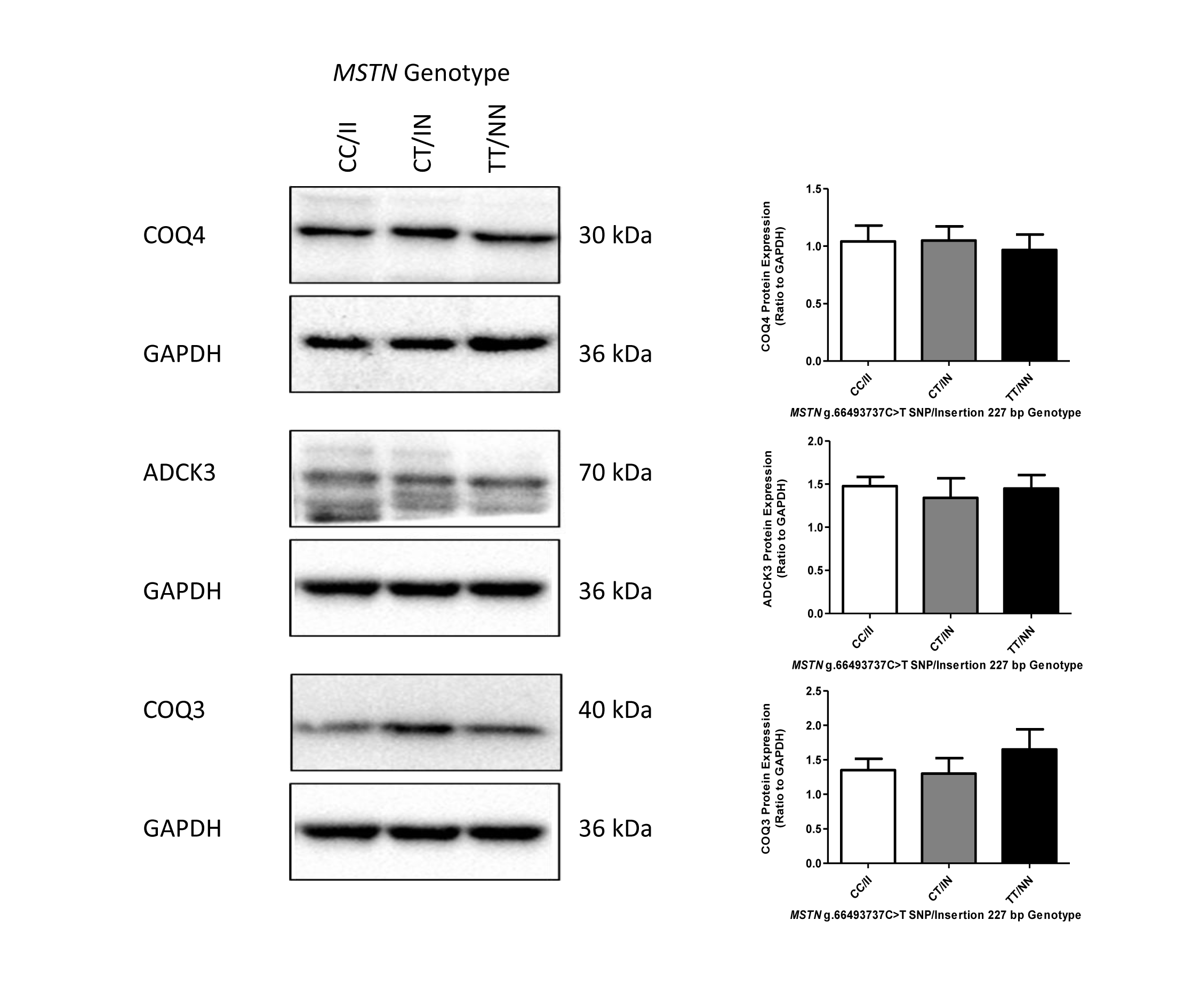

Supplement: S4 Fig — COQ4, ADCK3 and COQ3 protein levels were measured by western blot analysis in untrained Thoroughbred skeletal muscle protein lysates of three MSTN genotypes. A representative experiment out of six performed is shown for each protein. Densitometry was performed and corresponding results are presented with mean ± SEM, p-values where shown indicate significance as measured by a one-way ANOVA with a Bonferroni multiple comparison post-test. (TIF) [file pone.0186247.s004.tif]

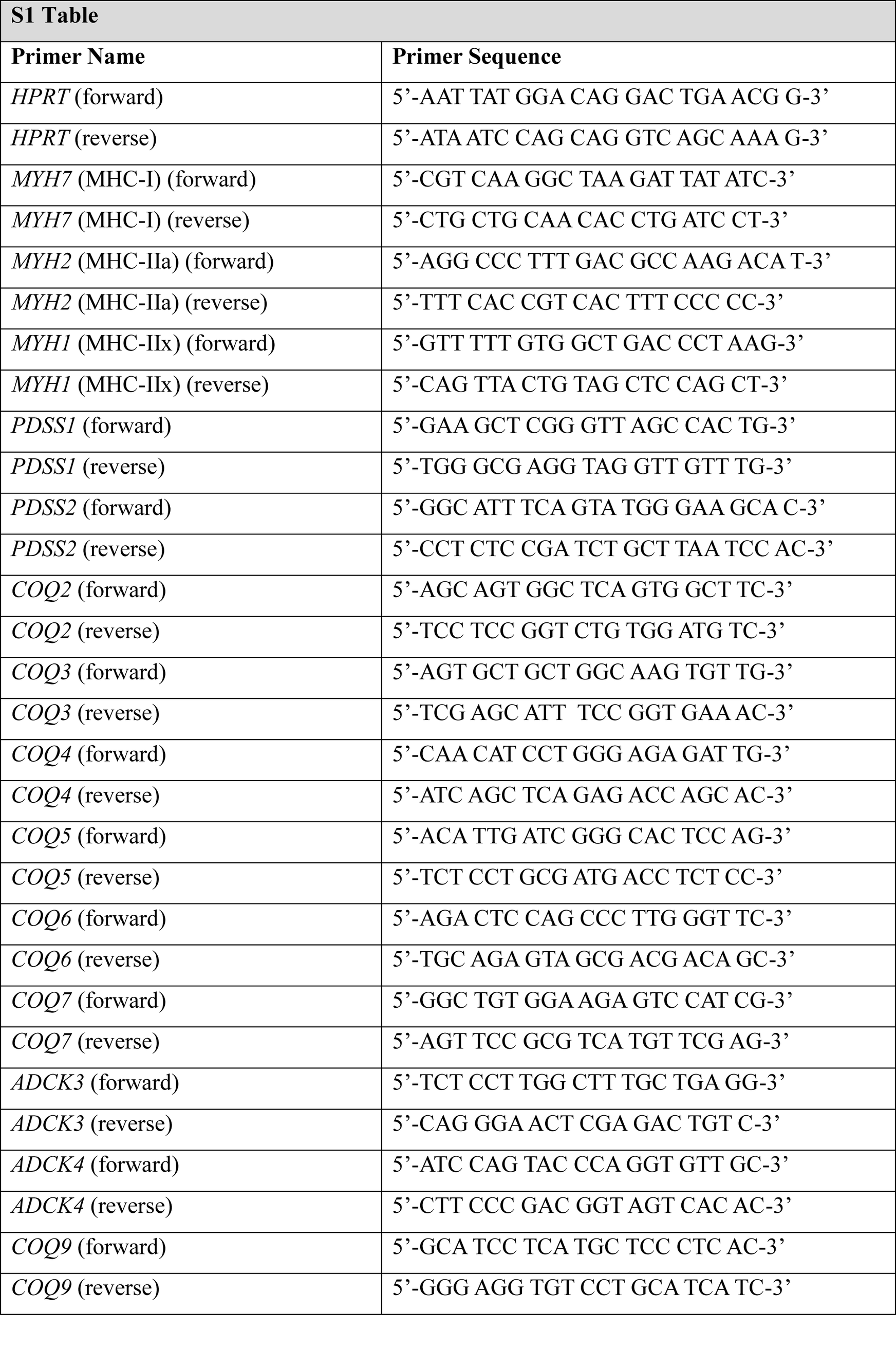

Supplement: S1 Table — (TIF) [file pone.0186247.s005.tif]

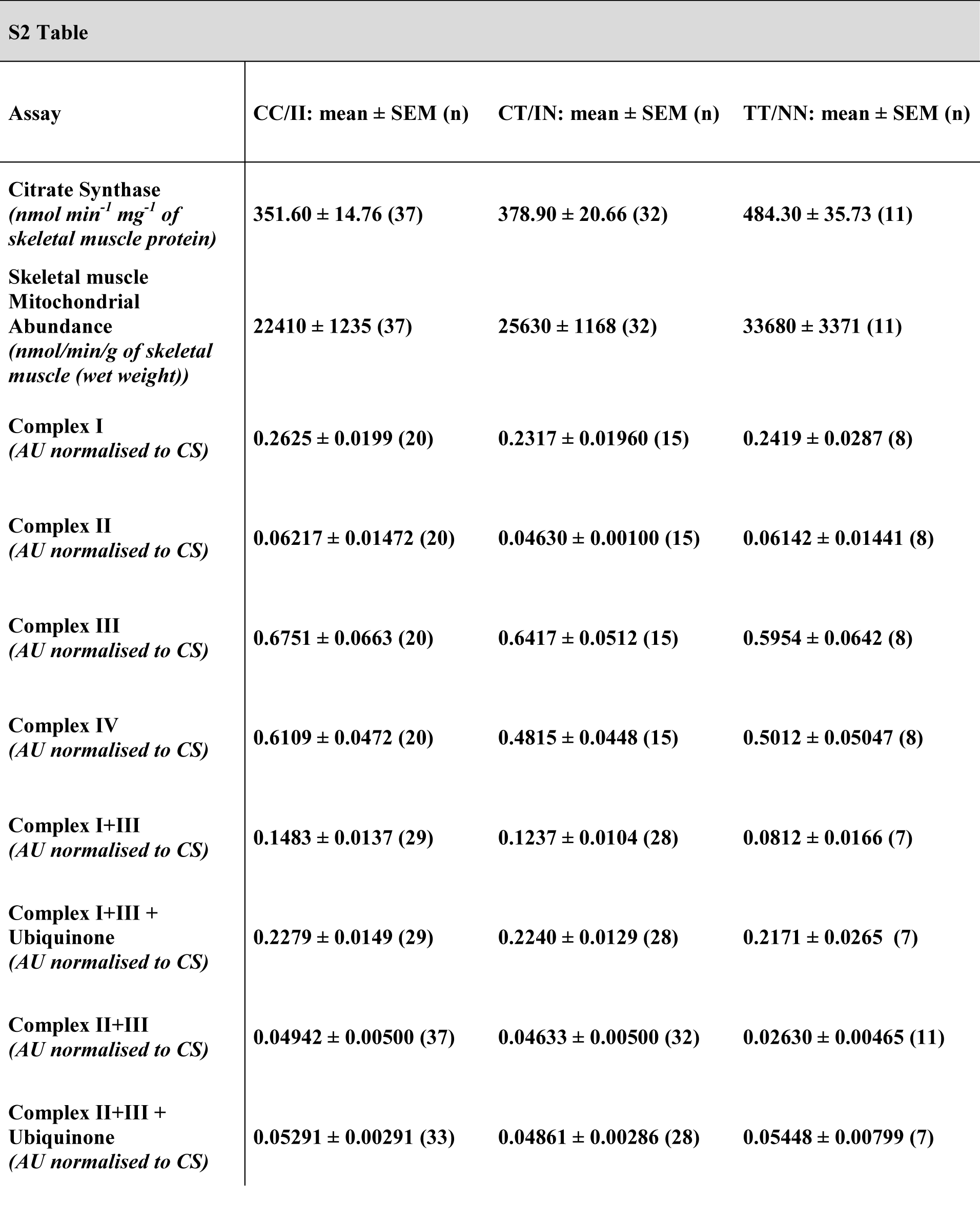

Supplement: S2 Table — Table of mean ± standard error of the mean (n) values for all spectrophotometric assays. Spectrophotometric results are based on experiments performed in at least duplicate for each sample. (TIF) [file pone.0186247.s006.tif]
